# Supramolecular complexes of dihydroazolopyrimidines with macrocyclic receptors: synthesis, rapid analysis, and evaluation

**DOI:** 10.3762/bjoc.22.122

**Published:** 2026-10-29

**Authors:** Daria Miasnikova, Vyacheslav Saraiev, Olena Shvets, Anna Korobka, Olena Chernyaeva, Yana Sakhno, Olga Vashchenko, Anna Shaposhnyk, Valentyn A Chebanov

**Affiliations:** 1 Institute of Functional Materials Chemistry, State Scientific Institution “Institute for Single Crystals” of National Academy of Sciences of Ukraine, 60 Nauky Ave., Kharkiv 61072, Ukraine; 2 Education and Research Institute of Chemistry, V. N. Karazin Kharkiv National University, 4 Svobody Sq., Kharkiv 61022, Ukraine; 3 Institute for Scintillation Materials, State Scientific Institution “Institute for Single Crystals” of National Academy of Sciences of Ukraine, 60 Nauky Ave., Kharkiv 61072, Ukraine

**Keywords:** dihydroazolopyrimidine, HPLC, macrocycle, solubilization study, supramolecular chemistry

## Abstract

One of the main challenges limiting the medical use of dihydroazolopyrimidine derivatives is their poor water solubility. However, this problem can be significantly reduced by binding to supramolecular receptors. In this study, we investigated the complexation of ethyl 5,7-dimethyl-4,7-dihydrotetrazolo[1,5-*a*]pyrimidine-6-carboxylate (DHP1) and ethyl 5-methyl-4,7-dihydro-[1,2,4]triazolo[1,5-*a*]pyrimidine-6-carboxylate (DHP2) with macrocyclic receptors: cucurbit[7]uril (CB7), β-cyclodextrin (β-CD), γ-cyclodextrin (γ-CD), and their modified derivatives, per-6-[(2-hydroxyethyl)amino]-6-deoxy-β-cyclodextrin (HEA-β-CD) and per-6-[(2-hydroxyethyl)amino]-6-deoxy-γ-cyclodextrin (HEA-γ-CD), in both solid and liquid phases. A quantitative high-performance liquid chromatography (HPLC) procedure for determining both DHPs in liquid systems was developed. The influence of time, temperature, and molar ratio CB7:DHPs on binding efficiency were systematically examined. Formation of the inclusion complexes was confirmed by thermogravimetric analysis (TGA) and powder X-ray diffraction (XRD) in the solid state and by HPLC in liquid media. It was demonstrated that the solubility of both DHPs in water increases through interaction with CB7. Furthermore, modification of CDs increases their binding affinity to DHP2.

## Introduction

Various nitrogen-containing heterocyclic compounds have attracted considerable attention due to their diverse biological activities [1–2]. Among these structures, tetrazole, triazole, and pyrimidine moieties [3] have been well explored as key structural motifs in various drug molecules, as they exhibit numerous biological activities, such as antimalarial [4], antifungal [5–6], antimicrobial [7–8], anticancer [9–10], anti-HBV [11], antitubercular [12–13], and α-glucosidase inhibitory activity [14]. A class of tetrazolopyrimidines has the properties of both pyrimidine and tetrazole scaffolds, as do triazolopyrimidines [15–18]. Among the derivatives of these heterocycles, compounds containing a dihydroazolopyrimidine fragment are also promising. Derivatives previously described in our publications were studied concerning their biological activity [19–20]: ethyl 5,7-dimethyl-4,7-dihydrotetrazolo[1,5-*a*]pyrimidine-6-carboxylate (DHP1), which showed antioxidant activity and ethyl 5-methyl-4,7-dihydro-[1,2,4]triazolo[1,5-*a*]pyrimidine-6-carboxylate (DHP2), which showed antimicrobial activity, making them interesting for further biological research.

One of the main limitations of using dihydroazolopyrimidine derivatives in the medical field is their low water solubility [21], which can be significantly improved by binding to supramolecular receptors [22–24]. In this research, we continue to study the influence of macrocyclic receptors on the properties of organic molecules [25–27]. Receptors of various origins, such as cyclodextrins, calixarenes and cucurbiturils (CBn, *n* = 5–8), have gained popularity in recent decades due to their high binding capacity for a wide range of compounds [28–29]. Despite their potential, many native macrocyclic receptors face limitations such as moderate binding affinity and poor selectivity. To overcome these challenges, the regioselective chemical modification has emerged as a key strategy to adjust the properties of the macrocyclic cavity. Specifically, the introduction of hydroxyethylamino groups onto the cyclodextrin scaffold not only enhances the aqueous solubility of the receptors but also provides additional binding sites, thereby potentially increasing the binding affinity and stability of the inclusion complexes [30].

Several studies are being conducted to identify and develop reliable supramolecular systems to improve the solubility and bioavailability of drugs and biologically active compounds. Therefore, the formation of inclusion complexes with macrocyclic receptors may be a promising strategy for overcoming solubility limitations, contributing to the expansion of pharmaceutical applications of these heterocyclic compounds for further biological and medical research.

This study focuses on the synthesis and characterization of supramolecular host–guest complexes formed by dihydroazolopyrimidines (DHPs) and the macrocyclic receptors cucurbit[7]uril (CB7), native cyclodextrins (CDs), and their modified derivatives, including per-6-[(2-hydroxyethyl)amino]-6-deoxy-β-cyclodextrin (HEA-β-CD) and per-6-[(2-hydroxyethyl)amino]-6-deoxy-γ-cyclodextrin (HEA-γ-CD).

The formation of the complexes was confirmed by thermogravimetric analysis (TGA), powder X-ray diffraction (XRD), and reversed-phase high-performance liquid chromatography (HPLC). An HPLC procedure was developed for the analysis of DHPs. The selection of chromatographic conditions, the ratio and type of mobile phases, and the influence of pH on optimal peak symmetry were evaluated. For quantitative analysis, a calibration curve was prepared, and the LOD and LOQ values were determined. Additionally, the binding of DHPs with CB7 in the liquid phase was studied to determine the binding constant and complexation efficiency.

**Figure 1:**
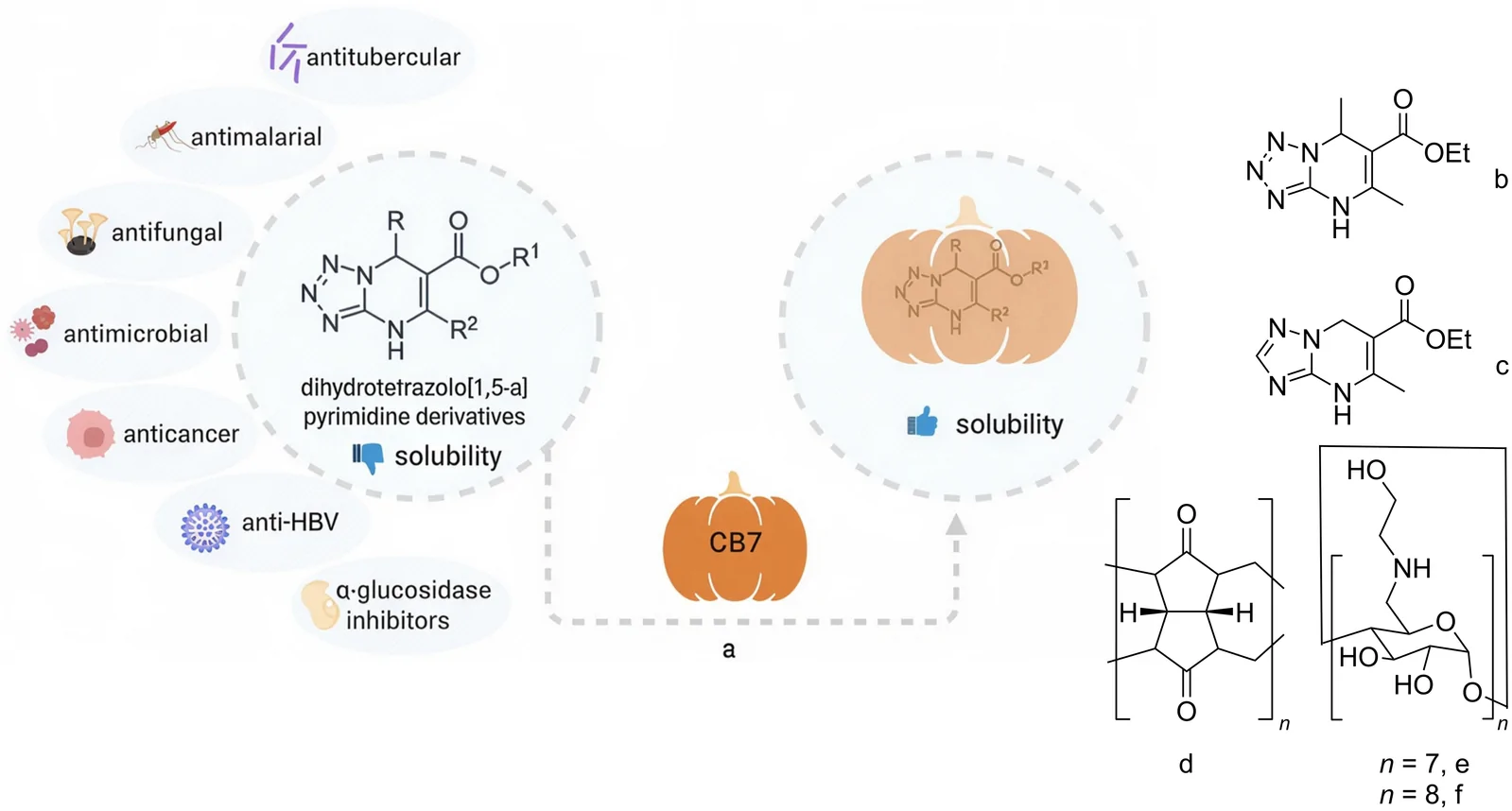
The schematic overview of the use of dihydroazolopyrimidine derivatives and the formation of complexes based on them (a); structures of the studied compounds: ethyl 5,7-dimethyl-4,7-dihydrotetrazolo[1,5-*a*]pyrimidine-6-carboxylate (DHP1) (b), ethyl 5-methyl-4,7-dihydro-[1,2,4]triazolo[1,5-*a*]pyrimidine-6-carboxylate (DHP2) (c), cucurbit[7]uril (CB7) (d); per-6-[(2-hydroxyethyl)amino]-6-deoxy-β-cyclodextrin (HEA-β-CD) (e) and per-6-[(2-hydroxyethyl)amino]-6-deoxy-γ-cyclodextrin (HEA-γ-CD) (f). Created in BioRender. Korobka, A. (2026) https://BioRender.com/mcwo5ot. This content is not subject to CC BY 4.0.

We focused on the less explored classes of dihydroazolopyrimidine derivatives and demonstrate the potential of CB7 to enhance their aqueous solubility, which is important for advancing the pharmaceutical utility of such heterocyclic compounds (Figure 1).

## Results and Discussion

### The development of a method for the analysis and determination of DHPs by HPLC

To study the binding between macrocyclic receptors and DHPs, firstly we developed the HPLC technique for the quantitative analysis of DHPs. The study of supramolecular systems in solution is challenging and requires careful selection of experimental conditions and analytical methods. Particular attention was given to adapting the methodology for different types of ligands and acceptors, as well as for systems with dynamic equilibriums. When developing the methodology, we considered its future use specifically for studying complex formation processes, focusing on the specifics of the interactions between system components, and the influence of solvent, temperature, and ionic strength of the medium. A series of experiments was performed under different conditions (see Supporting Information File 1, Table S1). Potassium dihydrogen phosphate (20 mM) was chosen as the main buffer system for one of the eluents. In all cases, chromatographic separation was carried out at an oven temperature of 35 °C, a flow rate of 1 mL min^−1^ and the injection volume of 1 μL.

**Figure 2:**
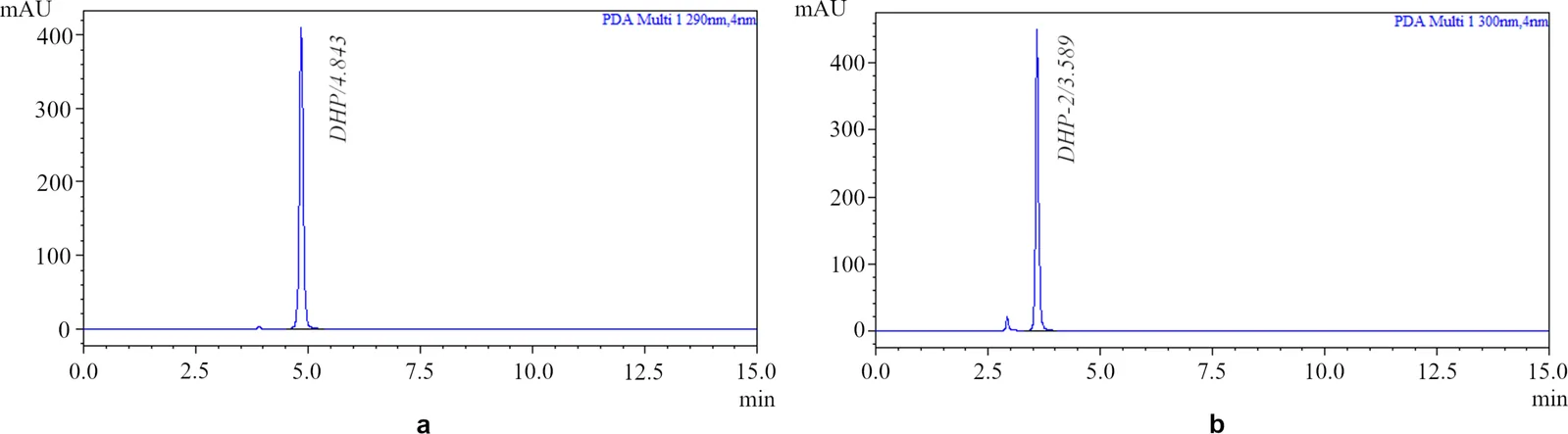
HPLC chromatograms of DHP1 (a) and DHP2 (b) in acetonitrile (Luna C18(2), Ø 4.6 × 250 nm, 1.0 mL min^−1^, 0–15 min, gradient 50% to 75% acetonitrile in a 20 mM phosphate buffer at pH 2.5, PDA: 290 nm for DHP1, 300 nm for DHP2).

Although methanol enables better separation, there is a risk of its “incorporation” (formation of solvate complexes) with the macrocycle (CB7). Isocratic elution resulted in poor separation between the main component and its impurities. Therefore, we selected conditions with a 15 min linear gradient from 50% to 75% acetonitrile in a 20 mM phosphate buffer at pH 2.5. Under these parameters, the DHP1 peak was observed at a retention time of approximately 4.8 min. These conditions resulted in sharp, symmetrical peaks with good separation from potential impurities, confirming the suitability of the method for quantitative analysis (Figure 2a). For DHP2 the same chromatographic conditions were applied and the DHP2 peak was observed at a retention time of approximately 3.6 min (Figure 2b). Furthermore, the method demonstrated potential applicability for analyzing structurally related compounds in future studies.

A calibration curve was constructed over the concentration range of 0.08–1.06 mg mL^−1^ for DHP1 (see Supporting Information File 1, Figure S1) and 0.05–0.85 mg mL^−1^ for DHP2 (see Supporting Information File 1, Figure S2), using standard solutions prepared from a stock solution in acetonitrile. The curve showed excellent linearity, indicating the reliability of the method for quantification. The coefficients of LOD (limit of detection) and LOQ (limit of quantitation) were determined according to the method described in [31]:


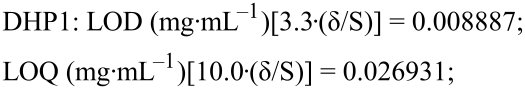



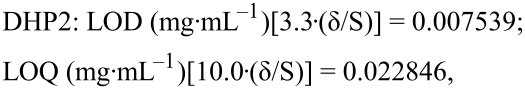


where δ is the standard deviation of regression line and S is the slope of the calibration curve.

### Synthesis and properties of CB7:DHP complexes in solid state

To obtain the CB7:DHP complexes in the solid state, we tested various synthesis and isolation conditions, with selected examples summarized in Table 1. To confirm actual complex formation, the *n*-hexane method is often used in literature [32–34]. This method is based on the fact that the macrocycle and its complexes are insoluble in *n*-hexane, while the free guest molecule is soluble. In our case, we used dichloroethane (DCE) instead of *n*-hexane due to the solubility of DHPs. After DCE washing and drying, we added DCE one more time, and only traces of DHPs were found. In contrast, when water was added to the DCE solutions, the complexes dissolved and DHPs dispersed preferentially in the organic phase rather than in water. To validate this control method for our study, a mechanical mixture of both components was prepared and analyzed. The analysis confirmed that the entire amount of DHPs mechanically mixed with CB7 was extracted into the DCE solution.

**Table 1:** Reaction conditions for obtaining CB7:DHPs complex.

Entry	synthesis conditions				*m* (DHP) [mg]	
	molar ratio of CB7 and DHP1	time	solvent	*T*_bath_ [°C]	free in DCE (HPLC)^a^	bound with CB7^b^

1	1:1	1 h	H_2_O/HCOOH	112	6.2	3.8
2	2:1	1 h	H_2_O/HCOOH	112	4.4	5.6
3	1:1	6 h	H_2_O/HCOOH	112	2.5	7.5
4	1:1	1 h	H_2_O	112	4.6	5.4
5	2:1	1 h	H_2_O	112	0.1	9.9
6	1:1	6 h	H_2_O	112	2.0	8.0
7	1:1	24 h	H_2_O/MeOH	60	5.2	4.8
8	2:1	24 h	H_2_O/MeOH	60	2.2	7.8

	molar ratio of CB7 and DHP2	time	solvent	*T*_bath_ [°C]	free in DCE (HPLC)	bound with CB7

9	1:1	1 h	H_2_O	112	4.4	5.6
10	2:1	1 h	H_2_O	112	3.0	7.0
11	1:1	5 h	H_2_O	112	0.2	9.8
12	2:1	5 h	H_2_O	112	0.1	9.9
13	1:1	24 h	H_2_O/MeOH	60	4.6	5.4
14	2:1	24 h	H_2_O/MeOH	60	0.1	9.9

^a^Mass of uncomplexed DHP determined quantitatively by HPLC following washing of each individual batch with DCE. ^b^Calculated mass of bound DHP.

As shown in Table 1, for the CB7:DHP1 system, reflux time affects binding in both H_2_O/HCOOH (entry 1/entry 3) and H_2_O (entry 4/entry 6). The highest product output was achieved in H_2_O after 1 hour of reflux with 2 equivalents of CB7 (Table 1, entry 5). For the CB7:DHP2 system, reflux time also affects binding efficiency. The highest product yield was obtained in H_2_O after 5 hours of reflux (Table 1, entry 11), while the difference between using 1 and 2 equivalents of CB7 in this solvent was negligible (entry 11/entry 12). High yields of the complex can also be achieved in a H_2_O/MeOH mixture at lower temperature, which, however, requires a reaction time of 24 h (Table 1, entry 14).

**Figure 3:**
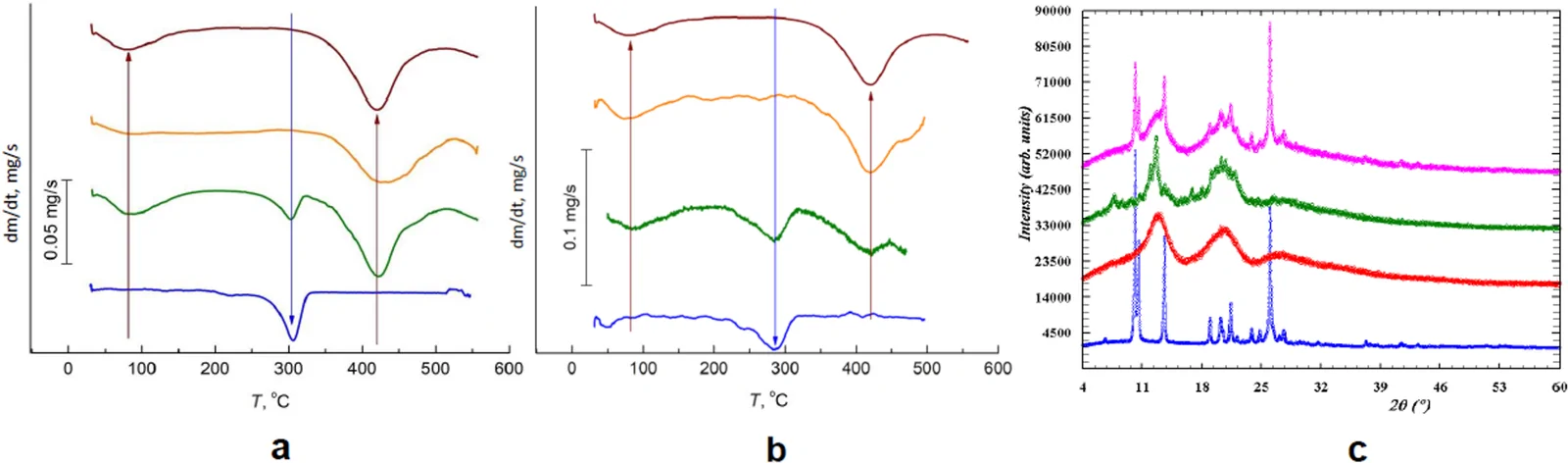
DTG thermograms of CB7 (brown), DHPs (blue), complex (orange), and mechanical 1:1 mixture (green): (a) CB7:DHP1, (b) CB7:DHP2, and (c) PXRD patterns of CB7:DHP2 complex (green), mechanical mixture (pink), DHP2 (blue), and CB7 (red).

Both complexes were analyzed using thermogravimetric analysis (TGA/DTG). The mixture for the analysis was prepared by grinding the starting reagents in a mortar in a 1:1 molar ratio. Comparison of the thermograms of the CB7:DHP1 (Figure 3a) and CB7:DHP2 (Figure 3b) complexes and the reagent mixtures clearly shows that the thermograms of the complexes, in contrast to that of the mixtures, do not contain the DTG peak of pure DHPs, confirming the formation of a stable complex. For the CB7:DHP2 system powder X-ray diffraction was also used to confirm binding (Figure 3c). The diffractogram of the complex does not contain DHP2 lines and the complex has some degree of crystallinity.

### Properties of CB7:DHP complexes in solution

The solubilization study is a well-known method to analyze the influence of a macrocyclic compound on the solubility of a guest in water [23,35–36]. First, we studied the influence of time on CB7:DHP1 binding. For this, three solutions were prepared, and aliquots were analyzed by HPLC after certain time intervals (Table 2).

**Table 2:** Time influence on the complexation of CB7 with DHP1.

Object	time interval				

	30 min	90 min	3 h	6 h	72 h

	amount of DHP1 in water (by HPLC) [%]^a^				

pure DHP1	29	34	34	35	38
1:1 molar ratio of CB7 and DHP1	47	60	61	66	69
2:1 molar ratio of CB7 and DHP1	58	74	77	79	89

^a^Values represent the relative percentage of DHP1 dissolved in the aqueous phase as determined by HPLC analysis at the indicated time intervals. Percentage values were utilized to clearly illustrate the time-dependent increase in apparent solubility relative to the total initial amount for each system.

As shown in Table 2, the amount of DHP1 dissolved in water after 90 minutes does not change noticeably over the following hours in both cases (molar ratios 1:1 and 2:1). However, the highest percentage of binding is reached 72 hours after the start of the mixing process, and no further significant change was observed after 72 h under the conditions studied. Since the amount of bound DHP1 after only 6 hours is relatively close to that after 72 hours, a shorter experimental duration may be sufficient for practical purposes. In the case of a 2:1 ratio, however, more time appears to be required to reach equilibrium due to the larger amount of CB7 in solution. A similar time-dependent behavior was observed for the CB7:DHP2 system (see Supporting Information File 1, Table S2). In this case, the binding efficiency also increases gradually with time, indicating comparable kinetic tendencies for both guest molecules.

Then we tested the influence of temperature on solubilization. The solution with a 1:1 molar ratio of CB7:DHP1 and the blank solution of pure DHP1 were placed in a water bath and stirred for 24 hours. The temperature was lowered every 3 hours until it reached 10 °C. At the end, the water bath was reheated to 20 °C. The samples were analyzed by HPLC at each temperature point (see Supporting Information File 1, Figure S3). The solubility of pure DHP1 increases linearly with temperature, which is typical for substances whose solubility rises as temperature increases due to higher dissolution energy. For the CB7:DHP1 complex, a steeper linear trend is observed, indicating that its solubility is more sensitive to temperature changes after CB7 is added. Therefore, CB7 enhances the solubility of DHP1.

**Figure 4:**
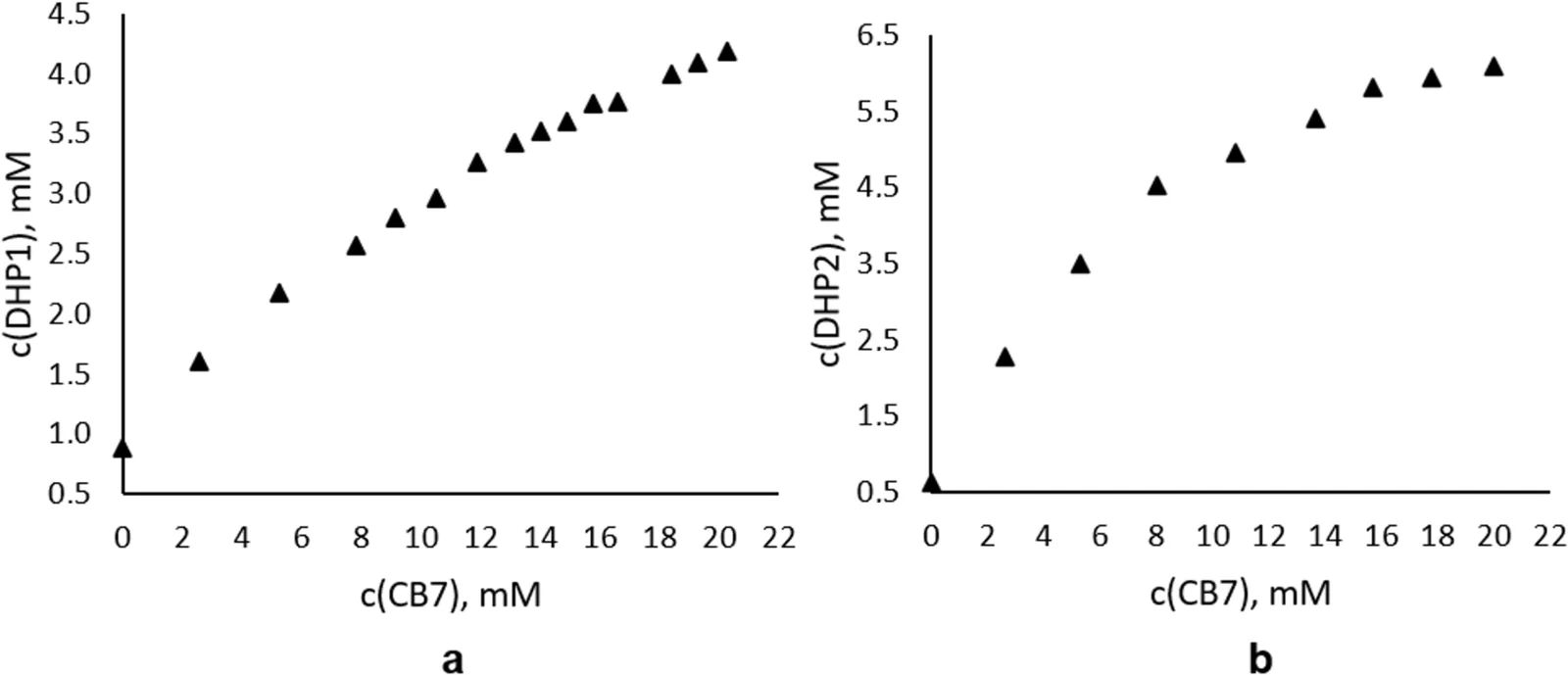
Phase-solubility diagrams of CB7:DHP1 (a) and CB7:DHP2 (b).

Since CB7 is known to enhance the aqueous solubility of poorly soluble compounds by host–guest complexation, we performed a phase-solubility study to evaluate its interaction with DHPs, focusing on changes in solubility and binding efficiency. To a solution containing a fixed amount of DHP1 or DHP2 portions of CB7 were added to achieve a concentration range of 2.6·10^−3^–2.2·10^−2^ M (Figure 4).

After solubilization, for CB7:DHP1 we obtained an A_L_-type curve (Figure 4a) indicating the solubility of the complex in water and, accordingly, the improvement of the solubility of DHP1 after binding to CB7. The A_L_-type curve indicates an interaction between the guest and host molecules with a molar ratio of 1:1 [35,37]. The value of *K*_1:1_ can be calculated according to Equation 1:


[1] $$\frac{\text{slope}}{S_{0}\cdot \left(1-\text{slope}\right)}=K_{1:1},$$

where *S*_0_ is the intrinsic solubility of the guest (i.e., the solubility in the aqueous medium when no CB7 is present), and slope is the slope of the linear (i.e., A_L_-type) phase-solubility diagram [38]. However, the value of *K*_1:1_ is very sensitive to small changes in *S*_0_, and for poorly soluble substances it can be difficult to obtain accurate *S*_0_ values. In addition, self-association of lipophilic guest molecules in aqueous media can lead to incorrect results. Considering these factors, it may be more accurate to determine the complexation efficiency (CE) using Equation 2:


[2] $$K_{1:1}\cdot S_{0}=\frac{\text{slope}}{1-\text{slope}}=\text{CE.}$$

The value of the CE depends only on the slope of the phase-solubility curve and smaller fluctuations are usually observed in CE values compared to the stability constant (*K*_1:1_). A CE of 0.1 suggests that one out of 11 macrocycle molecules forms a complex with the guest. With a CE of 0.01, only one out of 100 macrocycle molecules forms a complex [39]. We obtained *K*_1:1_ = 153.1 M^−1^ and CE = 0.18 (CE = [host]_complexed_/[host]_total_), so 18% of the host molecules are complexed, which means that 1 out of 5.56 (approx. 1 out of 6) CB7 molecules forms a bond with a DHP1 molecule. Complexation significantly enhanced the aqueous solubility of DHP1, rising from 0.198 mg mL^−1^ for the pure compound to 0.954 mg mL^−1^ at the end of the solubilization study.

For CB7:DHP2 an A_N_-type curve (Figure 4b) was obtained characterized by a negative deviation from linearity, indicating that the complex is soluble in water and that the solubility of DHP2 is enhanced upon binding to CB7. For calculation of the binding parameters, the linear portion of the phase solubility curve was used. The calculated association constant is *K*_1:1_ = 262.4 M^−1^ and CE = 0.39, so 39% of the host molecules are complexed, which means that approximately 1 out of 3 CB7 molecules interacts with a DHP2 molecule. Uncomplexed DHP2 exhibited a low solubility of 0.130 mg mL^−1^ in water, which increased nearly tenfold to 1.250 mg mL^−1^ following complexation.

**Figure 5:**
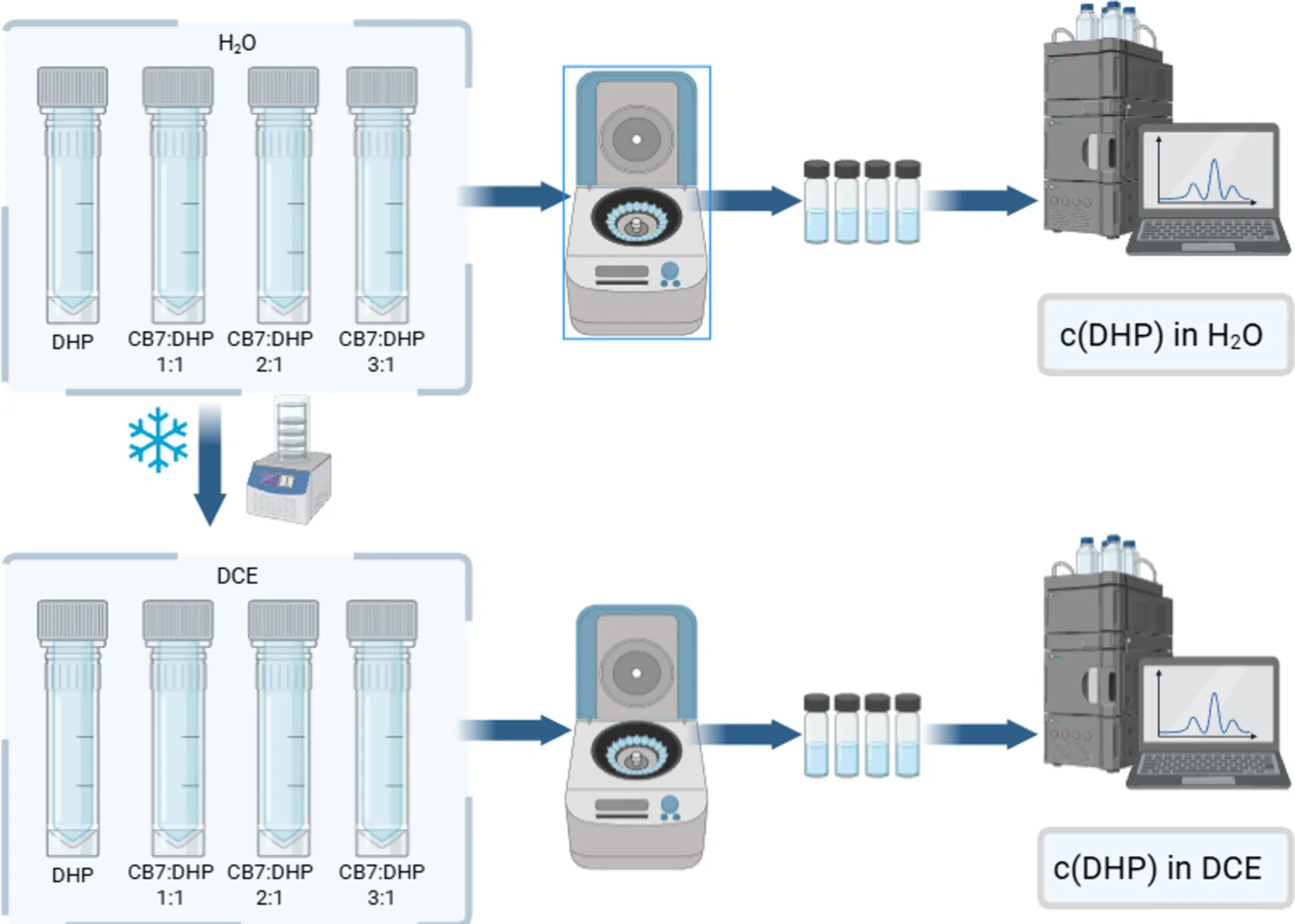
The schematic representation of the “solids/solutions” experiment. Created in BioRender. Korobka, A. (2026) https://BioRender.com/9e2g59p. This content is not subject to CC BY 4.0.

Finally, we combined the “solids/solutions” experiments: four solutions (pure DHP1, 1:2 molar ratio of CB7:DHP1, 1:1 molar ratio of CB7:DHP1, 2:1 molar ratio of CB7:DHP1, and 3:1 molar ratio of CB7:DHP1) were prepared in water, stirred, and analyzed by HPLC, then frozen and dried in a freeze dryer for 24 hours (Figure 5). A solution with a 4:1 molar ratio of CB7:DHP1 was also prepared, but CB7 did not fully dissolve; as this could influence the actual impact of CB7, we did not include this sample in our analysis. To the solid powders, 5 mL of DCE was added, and the samples were analyzed after 30 minutes of stirring. We obtain the same dependence as previously shown: in water the solubility of DHP1 increases with increasing amounts of CB7, in DCE solutions the amount of free DHP1 decreases, confirming binding (see Supporting Information File 1, Figure S4 and Figure S5).

Job’s method of continuous variation was used to determine the binding stoichiometry between CB7 and DHPs [40–41]. The UV–vis spectra for both DHPs were recorded at various host–guest ratios while maintaining a constant total concentration. Job’s plots were constructed by plotting Δ*A*·*х*_guest_ against *х*_guest_ (where Δ*A* is the change in absorption intensity during the experiment and *х*_guest_ is the mole fraction of the guest in each sample). The Job’s plot exhibited a clear maximum at a mole fraction of 0.5, indicating that both DHPs interact with CB7 in a 1:1 stoichiometric ratio during complex formation (see Supporting Information File 1, Figure S6).

### Cyclodextrin:DHP2 complexes

For DHP2, as it showed better binding affinity with CB7, our investigation was extended to include the more ubiquitous cyclodextrin family, specifically native β-CD and γ-CD. These macrocycles offer different cavity sizes, providing a basis for evaluating the spatial requirements of the DHP2 guest.

To ensure a direct comparison, the complexation reactions were conducted under the same synthetic conditions as those used for the CB7 complexes; the highest yield was achieved after 5 hours of reflux in H_2_O with a 1:1 molar ratio for both systems (Table 3). While the complexation of DHP2 with β-CD and γ-CD was successful, the resulting yields and binding performance were notably lower than those observed for the CB7:DHP2 system. Recognizing the limitations of native cyclodextrins in effectively binding DHP2, we sought to optimize the host–guest interaction through structural modification. The objective was to introduce functional groups capable of engaging in additional non-covalent interactions, such as hydrogen bonding or electrostatic attraction.

**Table 3:** Synthetic outcomes for CD:DHP2 complexation.

	free (DHP2) [%]	bound (DHP2) [%]

β-CD:DHP2	58	42
γ-CD:DHP2	56	44
HEA-β-CD:DHP2	14	86
HEA-γ-CD:DHP2	8	92

**Scheme 1:**
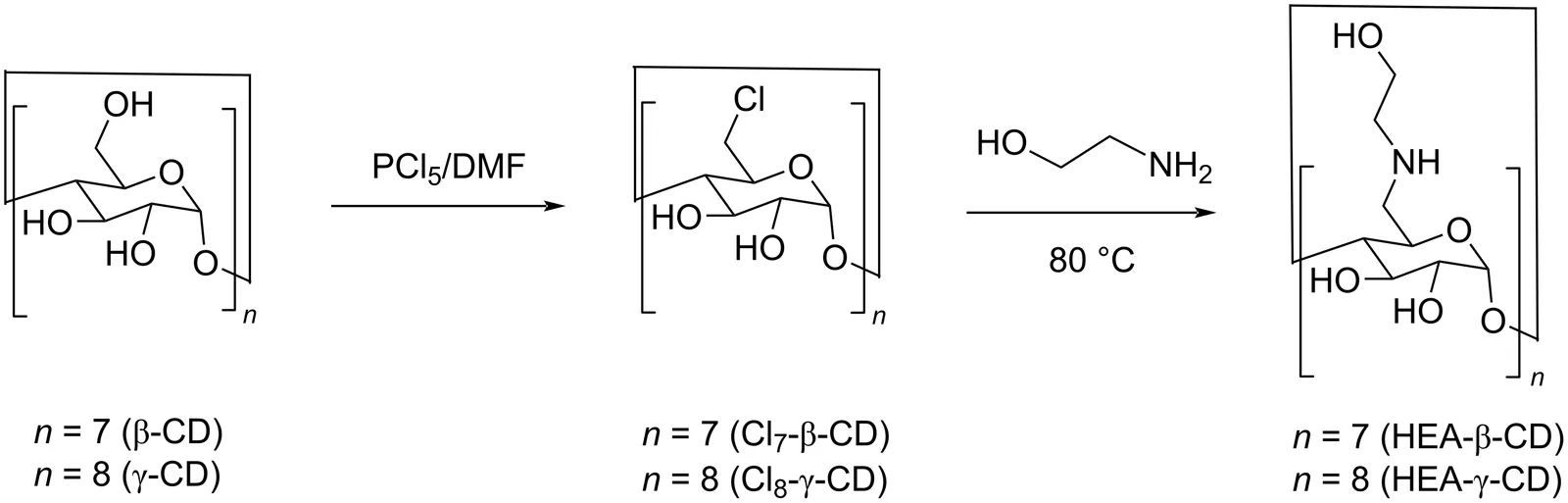
Synthetic route for the preparation of HEA-β-CD and HEA-γ-CD derivatives.

Therefore, we synthesized HEA-β-CD and HEA-γ-CD via a two-step procedure (Scheme 1). The synthetic route involved initial regioselective chlorination of the primary hydroxy groups, followed by nucleophilic substitution with monoethanolamine. This modification aimed to alter the physicochemical properties of the macrocycles and to evaluate how the introduction of hydroxyethylamino fragments influences the binding performance towards the DHP2 guest compared to native cyclodextrins.

The results obtained under identical conditions showed a significant increase in the amount of bound DHP2 for the modified cyclodextrins (Table 3). Furthermore, it was found that extending the reflux time to 12 hours promotes the quantitative binding of the guest molecule. This increase in the amount of bound DHP2 suggests that the HEA substituents do not merely act as passive structural modifications. Instead, they are likely to create a more favorable microenvironment for the guest, potentially by establishing additional hydrogen-bonding networks or through a "cap" effect that better isolates the DHP2 molecule within the hydrophobic cavity.

**Figure 6:**
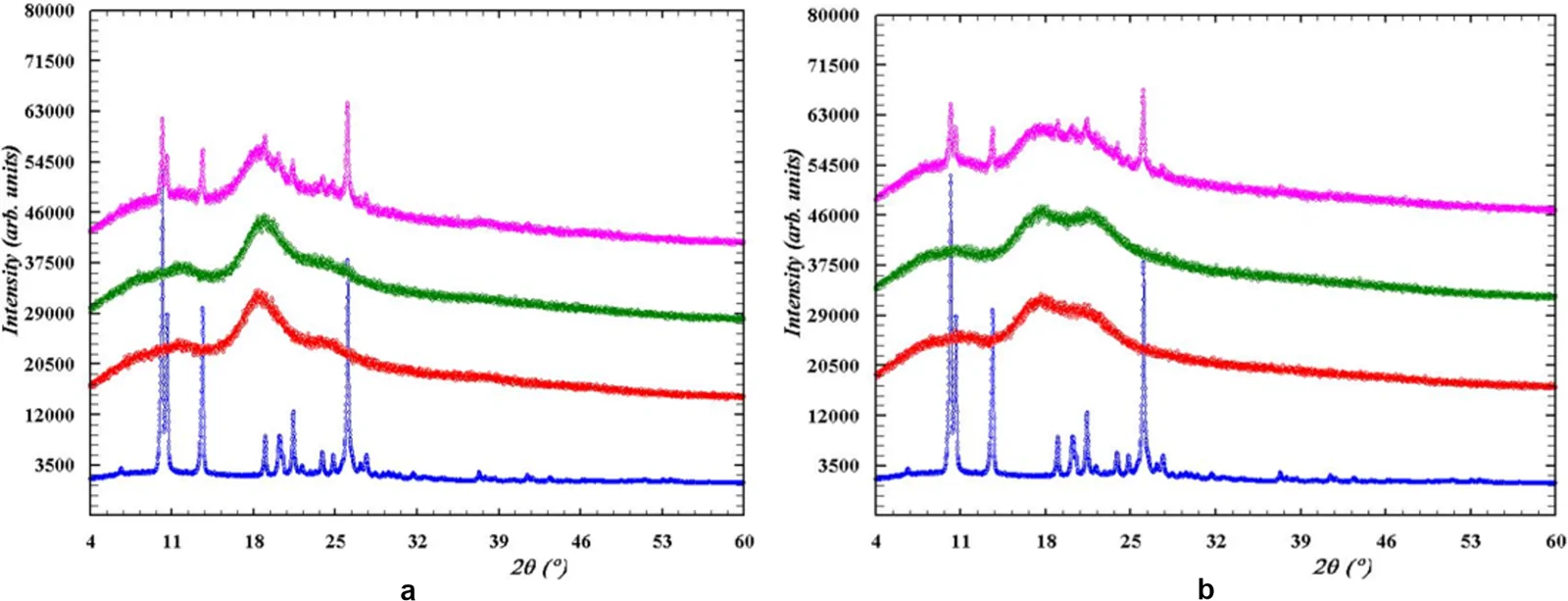
PXRD patterns of complexes (green), mechanical mixtures (pink), DHP2 (blue), macrocycle (red): HEA-β-CD (a), HEA-γ-CD (b).

The XRD analysis of the HEA-β-CD:DHP2 and HEA-γ-CD:DHP2 complexes revealed their amorphous nature (Figure 6). The complete disappearance of the guest’s crystalline signals in the powder patterns, in contrast to the physical mixtures where characteristic reflections of DHP2 remained visible, confirms the molecular dispersion of the substrate within the macrocyclic cavities and the successful formation of the inclusion complexes.

**Figure 7:**
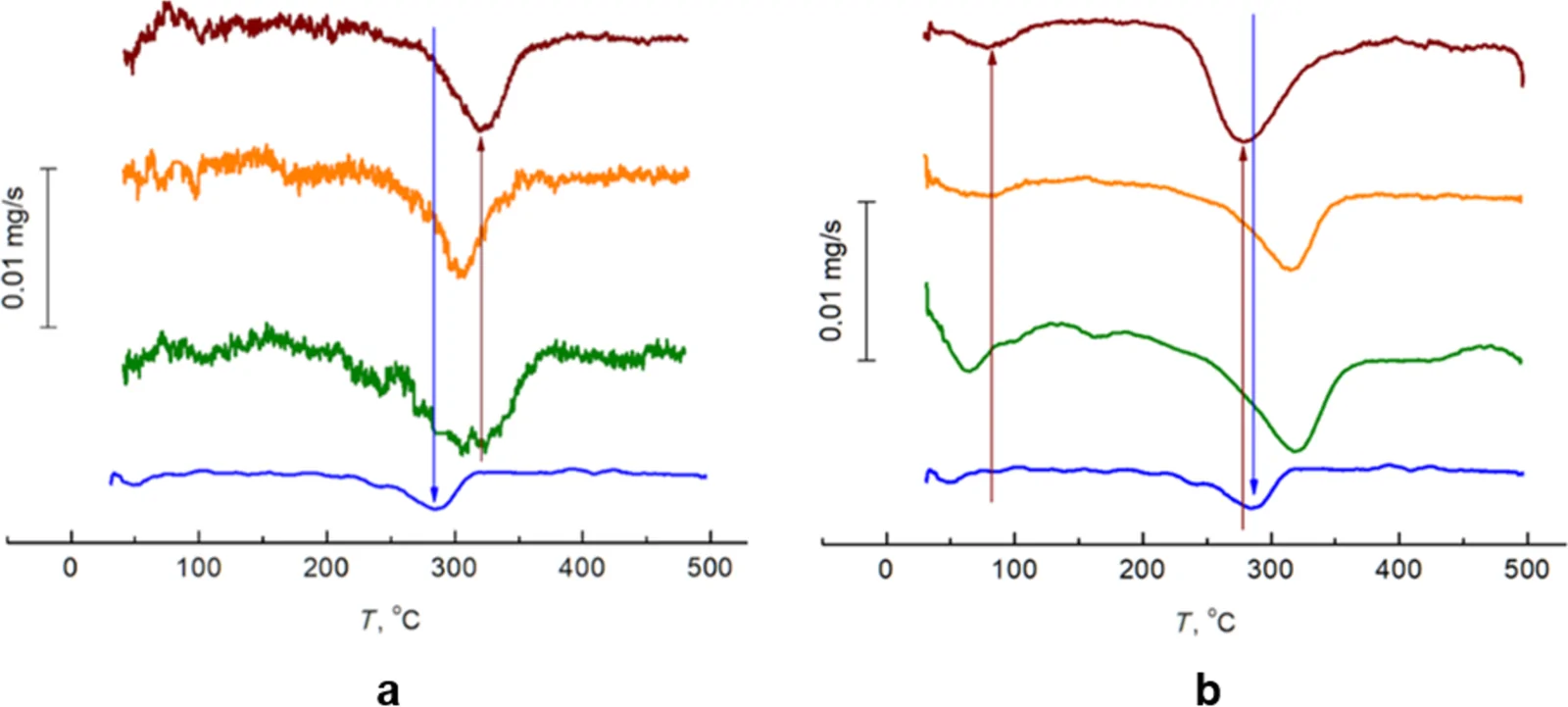
DTG thermograms of complexes (orange), mechanical mixtures (green), DHP2 (blue), macrocycle (brown): HEA-β-CD (a), HEA-γ-CD (b).

**Figure 8:**
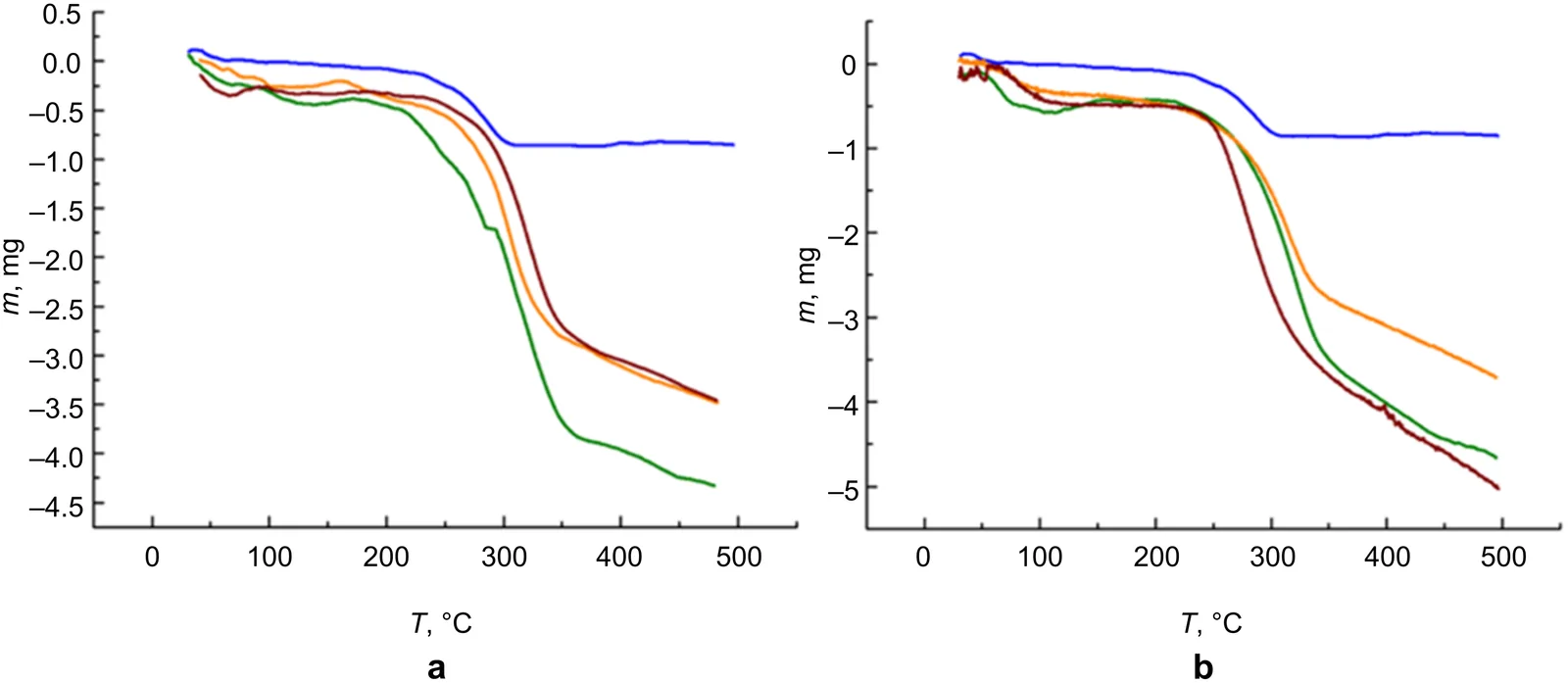
TGA thermograms of complexes (orange), mechanical mixtures (green), DHP2 (blue), macrocycle (brown): HEA-β-CD (a), HEA-γ-CD (b).

TGA and DTG data confirmed the formation of inclusion complexes between DHP2 and the modified receptors HEA-β-CD and HEA-γ-CD. The primary evidence for the creation of new supramolecular phases is the distinct change in the thermal degradation profiles: unlike mechanical mixtures, which exhibit broad and poorly defined decomposition intervals, the inclusion complexes show localized and well-defined mass-loss peaks (Figure 7). The narrowing of the degradation temperature range confirms that DHP2 molecules are encapsulated within the host cavities, where their stability is governed by the supramolecular system rather than the individual properties of the free guest. The thermal stability is significantly influenced by the macrocycle geometry, with HEA-β-CD providing maximum stabilization, as its degradation profile nearly duplicates that of the pure receptor (Figure 8a). This suggests high structural complementarity and efficient shielding of the guest within the β-CD cavity. In contrast, the HEA-γ-CD complex shows more intense mass loss in the guest’s degradation region (Figure 8b), likely due to its larger cavity diameter forming a less sterically constrained structure. Additionally, the altered dehydration profile in the 50–120 °C range (Figure 7) further confirms inclusion, resulting from the displacement of water molecules from the cyclodextrin cavities by the guest.

## Conclusion

This study demonstrates the potential of supramolecular complexes of cucurbit[7]uril to increase the water solubility and potentially the bioavailability of dihydroazolopyrimidine derivatives such as ethyl 5,7-dimethyl-4,7-dihydrotetrazolo[1,5-*a*]pyrimidine-6-carboxylate and ethyl 5-methyl-4,7-dihydro-[1,2,4]triazolo[1,5-*a*]pyrimidine-6-carboxylate.

An efficient HPLC-based procedure that provides high chromatographic accuracy for the identification and quantification of DHP-type compounds in aqueous and organic solvents has been developed. The described solvent system can be used for further studies of complexation with various macrocyclic receptors. The stability constant (*K*_1:1_), complexation efficiency (CE), and stoichiometry were determined for the CB7:DHP systems. Various methods for forming supramolecular complexes based on DHPs and CB7 in solid and liquid phases were developed. The possibility of separating mechanical mixtures from true complexes and the method for estimating solubilization parameters were presented. The temperature has been shown to affect the equilibrium between free molecules and complexes and an increase in temperature promotes the formation of more stable complexes. This also indicates that at certain temperatures, complexes can achieve greater stability, which is important for the development of delivery systems when using preparations in the form of ointments/suspensions or when performing medical physiotherapy procedures such as electrophoresis, ultrasound, laser therapy, and shock wave therapy.

The chemical modification of cyclodextrins with hydroxyethylamino groups proved to be an effective strategy for enhancing their complexing properties. The synthesized per-6-[(2-hydroxyethyl)amino]-6-deoxy-β-cyclodextrin and per-6-[(2-hydroxyethyl)amino]-6-deoxy-γ-cyclodextrin demonstrated significantly higher binding affinity toward the ethyl 5-methyl-4,7-dihydro-[1,2,4]triazolo[1,5-*a*]pyrimidine-6-carboxylate guest compared to native β-cyclodextrin and γ-cyclodextrin.

## Supporting Information

File 1 Materials, experimental part, NMR and MS spectra.

## Data Availability

Data generated and analyzed during this study is available from the corresponding author upon reasonable request.
