# Supplementary material for: Supramolecular complexes of dihydroazolopyrimidines with macrocyclic receptors: synthesis, rapid analysis, and evaluation
Source: Beilstein J Org Chem. 2026 Oct 29;22:1508–19. doi: 10.3762/bjoc.22.122 (PMC13725518; doi:10.3762/bjoc.22.122)
Supplement: Supplementary file 1 [file Beilstein_J_Org_Chem-22-1508-s001.pdf]

BEILSTEIN JOURNAL OF ORGANIC CHEMISTRY

Supporting Information

for

Supramolecular complexes of dihydroazolopyrimidines with macrocyclic receptors: synthesis, rapid analysis, and evaluation

Daria Miasnikova, Vyacheslav Saraiev, Olena Shvets, Anna Korobka, Olena Chernyaeva, Yana Sakhno, Olga Vashchenko, Anna Shaposhnyk and Valentyn A. Chebanov

Beilstein J. Org. Chem. **2026,** *22,* 1508–1519. doi:10.3762/bjoc.22.122

Materials, experimental part, NMR and MS spectra

License and Terms: This is a supporting information file under the terms of the Creative Commons Attribution License (https://creativecommons.org/licenses/by/4.0). Please note that the reuse, redistribution and reproduction in particular requires that the author(s) and source are credited and that individual graphics may be subject to special legal provisions.
The license is subject to the *Beilstein Journal of Organic Chemistry* terms and conditions: (https://www.beilstein-journals.org/bjoc/terms)

Table of contents

Materials and methods S2

Synthetic procedures S3

Dichloroethane wash procedure S7

Solubilization studies S8

HPLC determination of DHP S9

UV–vis analysis (Job`s plot) S9

HPLC conditions S10

NMR and MS spectra S14

References S19

Materials and methods

All reagents used were of analytical grade. Acetaldehyde (purity ≥99.5%), 5-aminotetrazole (5-ATZ) (purity ≥99.5%), ethyl 2-oxobutanoate (purity ≥99.5%), 3-amino-1,2,4-triazole (purity ≥99.5%), paraformaldehyde (purity ≥99.5%), ethyl acetoacetate (EAA) (purity ≥99.5%), dimethylformamide (DMF) (purity ≥99.5%) for the synthesis of DHP1 and DHP2, tetramethylammonium chloride (purity ≥98%), hydrochloric acid 36% *w*/*w* for obtaining CB7, phosphorous pentachloride (purity ≥98%), β-CD (purity ≥98%), γ-CD (purity ≥98), NaOH (purity ≥98%), monoethanolamine (purity ≥99%), ethanol (purity ≥99.5%) for the preparation of modified cyclodextrins, and dichloroethane (DCE) (purity ≥99.8%), acetonitrile (HPLC-grade, purity ≥99.9%), methanol (HPLC-grade, purity ≥99.9%), potassium dihydrogen phosphate (KH_2PO_4) (purity ≥99%), formic acid (HPLC-grade, purity ≥99%) were commercially available and used without additional purification.

The NMR spectra were recorded with an NMR spectrometer with a QUAD 400 C4 console and an Oxford magnet (400 MHz).

TGA/DTG thermograms were obtained by means of a Mettler TA 3000 thermoanalytical system. 7–8 mg of a sample was placed into an 80 μL aluminum oxide crucible with a pierced lid. The sample was heated in the temperature range of 25–300 °C at a scanning rate of 10 °C min^{-1} in an air atmosphere. The final mass of a sample was determined immediately after the end of the measurement and cooling the sample to room temperature. The scanning step was 0.17 °C.

ESIMS spectra were measured on a Shimadzu LCMS-2020 instrument (positive and negative ion mode, direct input of the sample). The spectra of DHPs were measured using a mobile phase (50:50, *v*/*v*): A = water, B = acetonitrile, while the spectra of CB7 were analyzed with mobile phase (50:50, *v*/*v*): A = water, B = 0.1% formic acid in acetonitrile.

The quantitative determination of DHPs was performed by reversed-phase high-performance liquid chromatography (HPLC). All HPLC experiments were carried out on a Shimadzu Prominence-i LC 2030C 3D Plus with a UV and PDA detector.

A Shimadzu UV-1700 PharmaSpec spectrometer was used to obtain the absorption spectra.

Powder X-ray diffractograms (XRD) were recorded using a Rigaku SmartLab diffractometer in Bragg–Brentano geometry (θ–θ scanning, Cu Kα radiation, Ni filter) in the angle range 2θ = 3–60° with a step of 0.01°.

Synthetic procedures

Ethyl 5,7-dimethyl-4,7-dihydrotetrazolo[1,5-*a*]pyrimidine-6-carboxylate (DHP1) was synthesized in a manner analogous to [1,2]. In a septum-sealed reaction vial, a solution of 5-aminotetrazole (0.14 g, 1.7 mmol), acetaldehyde (0.11 g, 2.5 mmol), and ethyl acetoacetate (0.22 g, 1.7 mmol) in water (3.5 mL) was irradiated in a microwave reactor at 100 °C for 35 min. The crystalline product started to precipitate during the reaction. The precipitate was filtered off, washed with water, and air-dried. Yield 88% (0.32 g).

Ethyl 5-methyl-4,7-dihydro-[1,2,4]triazolo[1,5-*a*]pyrimidine-6-carboxylate (DHP2) was synthesized in a manner analogous to [3]. A solution of 3-amino-1,2,4-triazole (0.14 g, 1.2 mmol), paraformaldehyde (0.04 g, 1.3 mmol), and ethyl acetoacetate (0.16 g, 1.2 mmol) in water (5 mL) was refluxed for 1 h. The resulting precipitate was filtered off, recrystallized from water, and air-dried. Yield 84% (0.29 g).

Cucurbit[7]uril (CB7) was synthesized in a manner analogous to [4] using tetraethylammonium chloride as template: glycoluril (1.42 g, 10 mmol), hydrochloric acid (36%, 7 mL), tetraethylammonium chloride (0.282 g, 1.7 mmol) were placed in a reaction flask (25 mL) and paraformaldehyde (0.6 g, 20 mmol) was added to the mixture. The reaction mixture was stirred at 95 °C for 2 hours and then dried on a rotary evaporator. Assuming that the reaction product contains 70% CB7 [5] and knowing its solubility in water [6], we separated insoluble homologs from the reaction mixture. The filtrate was evaporated until the precipitate formed, cooled and methanol (20 mL) was added until CB7 was completely precipitated. Yield 32% (0.53 g). M.p. > 300 °C.

^{1}H NMR (Figure S7) (400 MHz, 35wt% DCl in D_2O) δ 1.26 (d, 2H, CH_2), δ 2.46 (d, 2H, CH_2), δ 2.53 (s, 1H, CH).

MS-ESI (Figure S8) (m/z^+ (%)): 582.5 (100) $[M+2H]^{2+}$; MS-ESI (m/z^- (%)): 1207.4 (100) $[M+FA-H]^-$.

Per-6-deoxy-6-chloro-γ-cyclodextrin (Cl_8-γ-CD) and **per-6-deoxy-6-chloro-β-cyclodextrin (Cl_7-β-CD)** were synthesized in a manner analogous to [7].

Per-6-[(2-hydroxyethyl)amino]-6-deoxy-β-cyclodextrin (HEA-β-CD) and **per-6-[(2-hydroxyethyl)amino]-6-deoxy-γ-cyclodextrin (HEA-γ-CD)** were synthesized by reacting the corresponding chlorinated intermediates **Cl_7-β-CD** (1.0 g, 0.8 mmol) or **Cl_8-γ-CD** (1.0 g, 0.7 mmol) with an excess of monoethanolamine (0.5 g, 8.0 mmol or 0.43 g, 7.0 mmol, respectively) at 80 °C for 24 h. Upon completion of the reaction, the unreacted monoethanolamine was removed under reduced pressure. The resulting residue was precipitated into ethanol. The obtained solid was collected by filtration, washed successively with ethanol, and then dried in vacuum to constant weight.

HEA-β-CD: yield 80% (0.9 g).

^{1}H NMR (Figure S9) (400 MHz, DMSO-d_6) δ 2.54 – 2.69 (m, 14H), δ 2.80 (m, 7H) δ 2.91 (d, 7H), δ 3.28 – 3.43 (m, 28H), δ 3.64 (t, 7H), δ 3.72 (m, 7H), δ 4.87 (m, 7H), δ 5.75 (s, 14H).

^{13}C NMR (Figure S10) (100 MHz, DMSO-d_6) δ 49.1, δ 51.6, δ 60.2, δ 70.5, δ 72.5, δ 72.9, δ 83.2, δ 102.0.

MS-ESI (Figure S11) (m/z^+ (%)): 719.1 (100) $[M + 2H]^{2+}$, 1436.8 (27) $[M + H]^+$; MS-ESI (m/z^- (%)): 717.1 (54) $[M – 2H]^{2-}$, 1435.1 (100) $[M – H]^-$.

HEA-γ-CD: yield 85% (1.0 g).

^{1}H NMR (Figure S12) (400 MHz, DMSO-d_6) δ 2.52 – 2.67 (m, 16H), δ 2.78 (m, 8H), δ 2.89 (d, J = 12.1 Hz, 8H), δ 3.35 – 3.45 (m, 32H), δ 3.60 (t, 8H), δ 3.68 (m, 8H), δ 4.92 (d, 8H), δ 5.83 (s, 16H).

^{13}C NMR (Figure S13) (100 MHz, DMSO-d_6) δ 49.2, δ 51.7, δ 60.2, δ 70.7, δ 72.7, δ 72.7, δ 82.7, δ 101.9.

MS-ESI (Figure S14) (m/z^+): 548.1 (27) $[M + 3H]^{3+}$, 821.7 (100) $[M + 2H]^{2+}$.

Synthesis of CB7 complex with DHPs.

Method A (H_2O or H_2O/HCOOH): the reagents in a 1:1 (52 mg, 0.045 mmol of CB7; 10 mg; 0.045 mmol of DHP1) or 2:1 (104 mg; 0.090 mmol of CB7; 10 mg, 0.045 mmol of DHP1) (CB7:DHP1) molar ratio were dissolved in a solvent (1 mL of H_2O/HCOOH (50:50 vol %) or 10 mL of H_2O), refluxed for 1 hour (or 6 hours). The reagents in a 1:1 (56 mg, 0.048 mmol of CB7; 10 mg; 0.048 mmol of DHP2) or 2:1 (112 mg, 0.096 mmol of CB7; 10 mg; 0.048 mmol of DHP2) molar ratio were dissolved in a 5 mL of H_2O, refluxed for 1 hour (or 5 hours). Then the solvent was evaporated on a rotary evaporator (T_{bath}= 65–75 °C) and dried under vacuum for 10 hours.

Method B (H_2O/MeOH): the amount of CB7, corresponding to 1:1 (52 mg, 0.045 mmol of CB7 for DHP1, 52 mg, 0.045 mmol of CB7 for DHP2) and 2:1 (104 mg; 0.090 mmol of CB7 for DHP1, 112 mg, 0.096 mmol of CB7 for DHP2) molar ratio, was dissolved in water (5 mL). DHP1 (10 mg; 0.045 mmol) (or DHP2 (10 mg; 0.048 mmol)) was added, and then methanol (1.5 mL) was also added to dissolve DHP. The solutions became slightly cloudy, probably due to the precipitation of CB7. Solutions were left overnight in an open flask (T_{bath}= 60 °C). According to the authors of the article [8], the gradual evaporation of methanol in which DHPs are dissolved contributes to their solubilization in the aqueous solution of CB7. Afterwards, a precipitate was observed in the 1:1 solution, while the 2:1 solution was transparent. Both solutions were then cooled to room temperature, frozen, and dried in a freeze dryer for 20 hours.

CB7:DHP1: (for optimized conditions (entry 5)) yield 99% (113.0 mg).

^{1}H NMR (Figure S15) (400 MHz, 35wt% DCl in D_2O) δ -1.84 (t, 3H, CH_3), δ -1.49 (d, 3H, CH_3), δ -0.72 (s, 3H, CH_3), δ 1.11 (q, 2H, CH_2), δ 1.27 (d, 2H, CH_2), δ 2.49 (d, 2H, CH_2), δ 2.55 (s, 1H, CH), δ 2.78 (q, 1H, CH).

CB7:DHP2:(for optimized conditions (entry 11)) yield 98 % (65.0 mg).^{1}H NMR (Figure S16) (400 MHz, 35wt% DCl in D_2O) δ -1.86 (s, 3H, CH_3), δ -0.75 (s, 3H, CH_3), δ 1.07 (s, 2H, CH_2), δ 1.27 (d, 2H, CH_2), δ 1.85 (s, 2H, CH_2), δ 2.48 (d, 2H, CH_2), δ 2.54 (s, 1H, CH), δ 5.36 (s, 1H, CH).

Synthesis of CD complexes with DHP2. DHP2 (10.0 mg, 0.048 mmol) and the corresponding macrocycle (β-CD (54.5 mg, 0.048 mmol), γ-CD (62.3 mg, 0.048 mmol), HEA-β-CD (69.0 mg, 0.048 mmol), HEA-γ-CD (79.0 mg, 0.048 mmol)) in a 1:1 molar ratio were dissolved in 5 mL of water and refluxed with stirring for 1 or 5 hours. Then the solvent was evaporated on a rotary evaporator (T_{bath}= 65–75 °C) and dried under vacuum for 10 hours.

HEA-β-CD:DHP2: yield 86% (68 mg).

^{1}H NMR (Figure S17) (400 MHz, DMSO-d_6) δ 1.22 (t, 3H, CH_3), δ 2.31 (s, 3H, CH_3), δ 2.63 – 2.66 (m, 14H), δ 2.81 (m, 7H), δ 2.97 (d, 7H), δ 3.34 (m, 28H), δ 3.62 (t, 7H), δ 3.73 (m, 7H), δ 4.11 (q, 2H, CH_2), δ 4.81 (s, 2H, CH_2), δ 4.87 (m, 7H), δ 5.78 (s, 14H), δ 7.71 (s, 1H, CH).

HEA-γ-CD:DHP2: yield 92% (81.9 mg).

^{1}H NMR (Figure S18) (400 MHz, DMSO-d_6) δ 1.22 (t, 3H, CH_3), δ 2.31 (s, 3H, CH_3), δ 2.58 (m, 16H), δ 2.76 (m, 8H), δ 2.86 (d, 8H), δ 3.58 (s, 8H), δ 3.66 (m, 8H), δ 4.10 (q, 2H, CH_2), δ 4.81 (s, 2H, CH_2), δ 4.89 (m, 7H), δ 5.83 (s, 16H), δ 7.71 (s, 1H, CH).

All obtained complexes were tested using the **“**dichloroethane wash procedure” described below.

Dichloroethane wash procedure

The true complexation was confirmed by the dichloroethane (DCE) wash procedure and subsequent determination of the non-complexed DHPs by HPLC analysis [9-11]. The solubility of DHPs in different solvents was preliminarily studied and DCE was selected as the optimal solvent. The obtained complexes were dissolved in an appropriate volume of DCE (10 mL) and stirred for 30 min at 150 rpm. Then, 1 mL of the solution was sampled, centrifuged for 1 min at 3500 rpm and analyzed by HPLC to determine the amount of DHP1 (or DHP2) that did not bind to CB7. The solution was then filtered to remove the dissolved free DHP1 (or DHP2) and dried to obtain the complexes.

Solubilization studies

10 mg ($4.5\cdot10^{-3}$ M) of DHP1 was dissolved in 10 mL of H_2O (T_{bath}= 25 °C) and portions of CB7 (10 mg) were added every 2 hours ($2.6\cdot10^{-3}$ to $2.2\cdot10^{-2}$ M). 20 mg ($9.6\cdot10^{-3}$ M) of DHP2 was dissolved in 10 mL of H_2O (T_{bath}= 25 °C) and portions of CB7 (30 mg) were added every 2 hours ($2.6\cdot10^{-3}$ to $2.2\cdot10^{-2}$ M). The samples were taken, centrifuged for 1 min at 3500 rpm, and analyzed by HPLC.

Time influence on CB7:DHPs binding. Three solutions were prepared in 20 mL of H_2O: pure DHP or DHP2; 1:1 molar ratio of CB7:DHP1 (or DHP2); 2:1 molar ratio of CB7:DHP1 (or DHP2). Then the vials equipped with magnetic stirring bars were placed on the stirrer (T_{bath}= 25 °C) and aliquots (0.2 mL) were taken (after 30 min, 90 min, 3 h, 6 h, 72 h), centrifuged for 1 min at 3500 rpm, and analyzed by HPLC.

Influence of temperature on CB7:DHP1 binding. Two solutions were prepared in glass vials in 20 mL of H_2O: pure DHP1 (10 mg) and a 1:1 molar ratio of CB7:DHP1 (10 mg of DHP1, 52 mg of CB7). The vials equipped with magnetic stirring bars were placed on a stirrer and stirred at T_{bath}= 40 °C for 24 hours, aliquots were taken, centrifuged for 1 min at 3500 rpm and analyzed by HPLC. Then the temperature was lowered to 30 °C, stirred for 3 hours, samples were taken, centrifuged for 1 min at 3500 rpm and analyzed by HPLC. The same procedure was carried out at 25 °C, 20 °C and 10 °C. Finally, the temperature was raised again to 20 °C.

Solubilization study with subsequent isolation of the solid product. Four solutions were prepared in glass vials (7 mL of H_2O; T_{bath}= 25 °C): DHP1 (5 mg); 1:2 molar ratio of CB7:DHP1 (5 mg DHP1, 13 mg CB7); 1:1 molar ratio of CB7:DHP1 (5 mg DHP1, 26 mg CB7); 2:1 molar ratio of CB7:DHP1 (5 mg DHP1, 52 mg CB7); 3:1 molar ratio of CB7:DHP1 (5 mg DHP1, 78 mg CB7). The vials equipped with magnetic stirring bars were placed on the stirrer and stirred for 48 hours, centrifuged for 5 min at 3500 rpm, samples were taken, and analyzed by HPLC. The samples were then frozen and dried

in a freeze dryer for 24 hours. 5 mL of DCE was added, stirred for 30 min, centrifuged for 5 min at 3500 rpm and analyzed by HPLC for the amount of DHP1 that not bound to the complex. The solutions were filtered to remove free DHP1 and dried to obtain the complex.

HPLC determination of DHP

The selection of optimal conditions, mobile phases, elution modes, and chromatographic columns is described in the Results and Discussion section; these are the most optimal among those tested. According to this method, separation was performed on a Luna C18(2) column (4.6 mm × 250 mm, 5 μm) using 20 mM potassium dihydrogen phosphate (pH 2.50) as solvent A and acetonitrile as solvent B at a flow rate of 1 mL min^{-1}. The gradient was as follows: 0–2.0 min, 50% B, 7.0 min, 75% B, hold for 3 min, 12.0 min, 50% B, hold for 3 min. The injection volume was 1 μL, the oven temperature was 35 °C, and the detection wavelength was 290 nm for DHP1 and 300 nm for DHP2. The DHPs stock solutions were prepared at concentrations of 2.12 mg mL^{-1} for DHP1 and 1.03 mg mL^{-1} for DHP2. These solutions were further diluted with acetonitrile (HPLC grade) to 0.08–1.06 mg mL^{-1} for DHP1 and 0.05–0.85 mg mL^{-1} for DHP2; the calibration curves were prepared.

UV–vis analysis (Job`s plot)

The stock solutions of DHP1 ($4.48 \cdot 10^{-4}$ M), DHP2 ($7.2 \cdot 10^{-4}$ M) and CB7 ($4.48 \cdot 10^{-4}$ M and $7.2 \cdot 10^{-4}$ M) were prepared in H_2O. A series of solutions were then prepared with a constant total concentration of DHP1 (or DHP2) and CB7, while systematically varying their molar fractions from 0.1 to 0.9. The solutions were stirred for 2 hours, then diluted (fourfold for DHP2, twofold for DHP1). 3 mL of the probe solution was placed in a quartz cuvette with a 1 cm optical path length. The absorbance values for calculations were taken at λ = 294 nm for DHP1 and λ = 306 nm for DHP2. Different

detection wavelengths were selected based on the distinct UV–vis absorption maxima of each compound, maximizing sensitivity and selectivity for each individual analyte.

HPLC conditions

Table S1: HPLC procedures: testing the condition for DHP1 analysis.

Method	Column	Run time, min	Start % eluent A	Start % eluent B	Gradient	
					Time (min)	% eluent B
1	Luna C18(2) 4.6 mm × 250 mm, 5 μm	15	50%; 20mM pH 2.50 potassium dihydrogen phosphate	50%; Acetonitrile	0.01<br>2.00<br>7.00<br>10.00<br>12.00<br>15.00	50<br>50<br>75<br>75<br>50<br>50
2	Luna C18(2) 4.6 mm × 250 mm, 5 μm	15	50%; 20mM pH 2.50 potassium dihydrogen phosphate	50%; Methanol	0.01<br>2.00<br>7.00<br>10.00<br>12.00<br>15.00	50<br>50<br>75<br>75<br>50<br>50
3	Luna C18(2) 4.6 mm × 250 mm, 5 μm	10	25%; 20mM pH 2.50 potassium dihydrogen phosphate	75%; Acetonitrile	-	
4	Luna C18(2) 4.6 mm × 250 mm, 5 μm	10	25%; 20mM pH 2.50 potassium dihydrogen phosphate	75%; Methanol	-	
5	Shim-pack C18 4.6 mm × 250 mm, 5 μm	15	50%; 20mM pH 2.50 potassium dihydrogen phosphate	50%; Methanol	0.01<br>2.00<br>7.00<br>10.00<br>12.00<br>15.00	50<br>50<br>75<br>75<br>50<br>50
6	Shim-pack C18 4.6 mm × 250 mm, 5 μm	15	50%; 20mM pH 7.20 potassium dihydrogen phosphate	50%; Acetonitrile	0.01<br>2.00<br>7.00<br>10.00<br>12.00<br>15.00	50<br>50<br>75<br>75<br>50<br>50
7	Kinetex F5 4.6 mm × 250 mm, 5 μm	15	50%; 20mM pH 2.50 potassium dihydrogen phosphate	50%; Acetonitrile	0.01<br>2.00<br>7.00<br>10.00<br>12.00<br>15.00	50<br>50<br>75<br>75<br>50<br>50

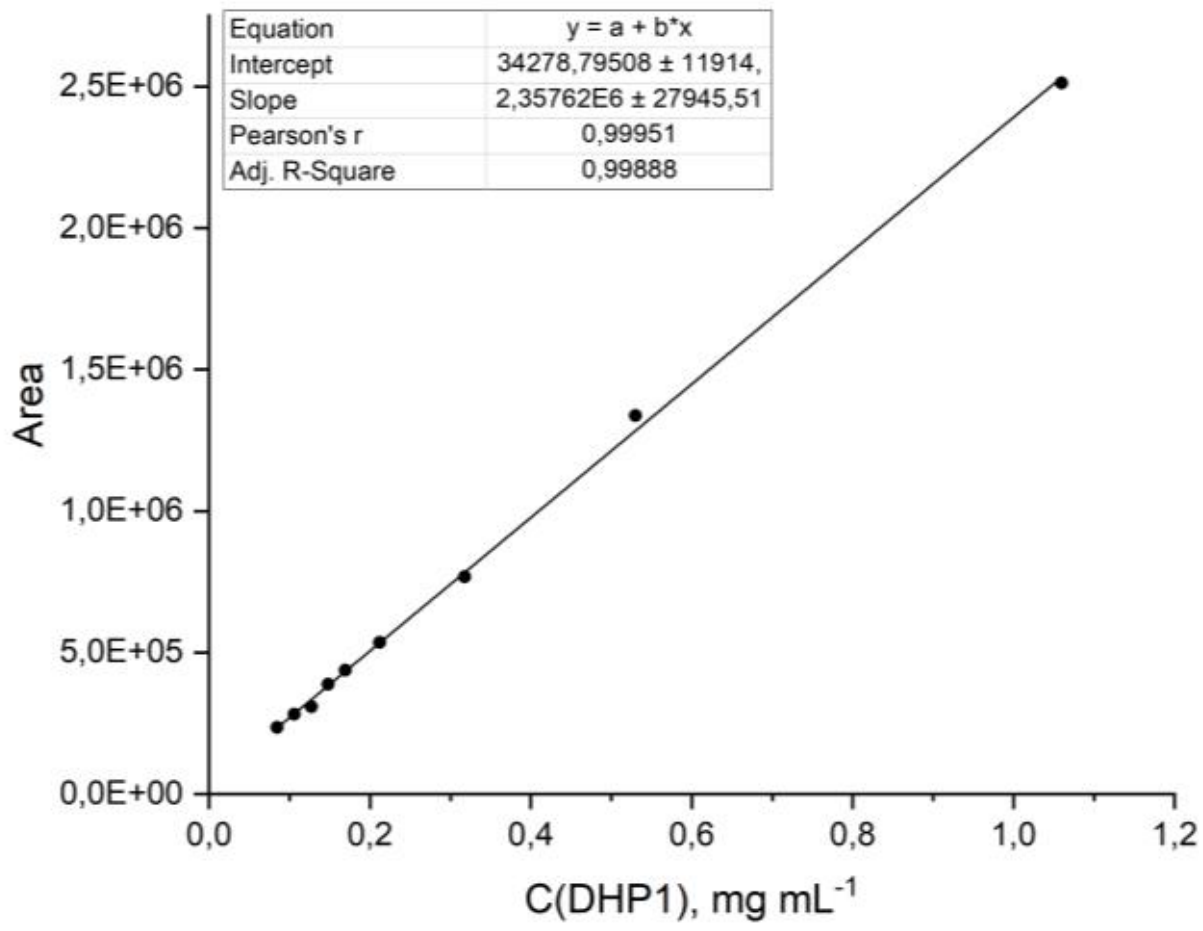

Figure S1: Calibration curve for DHP1.

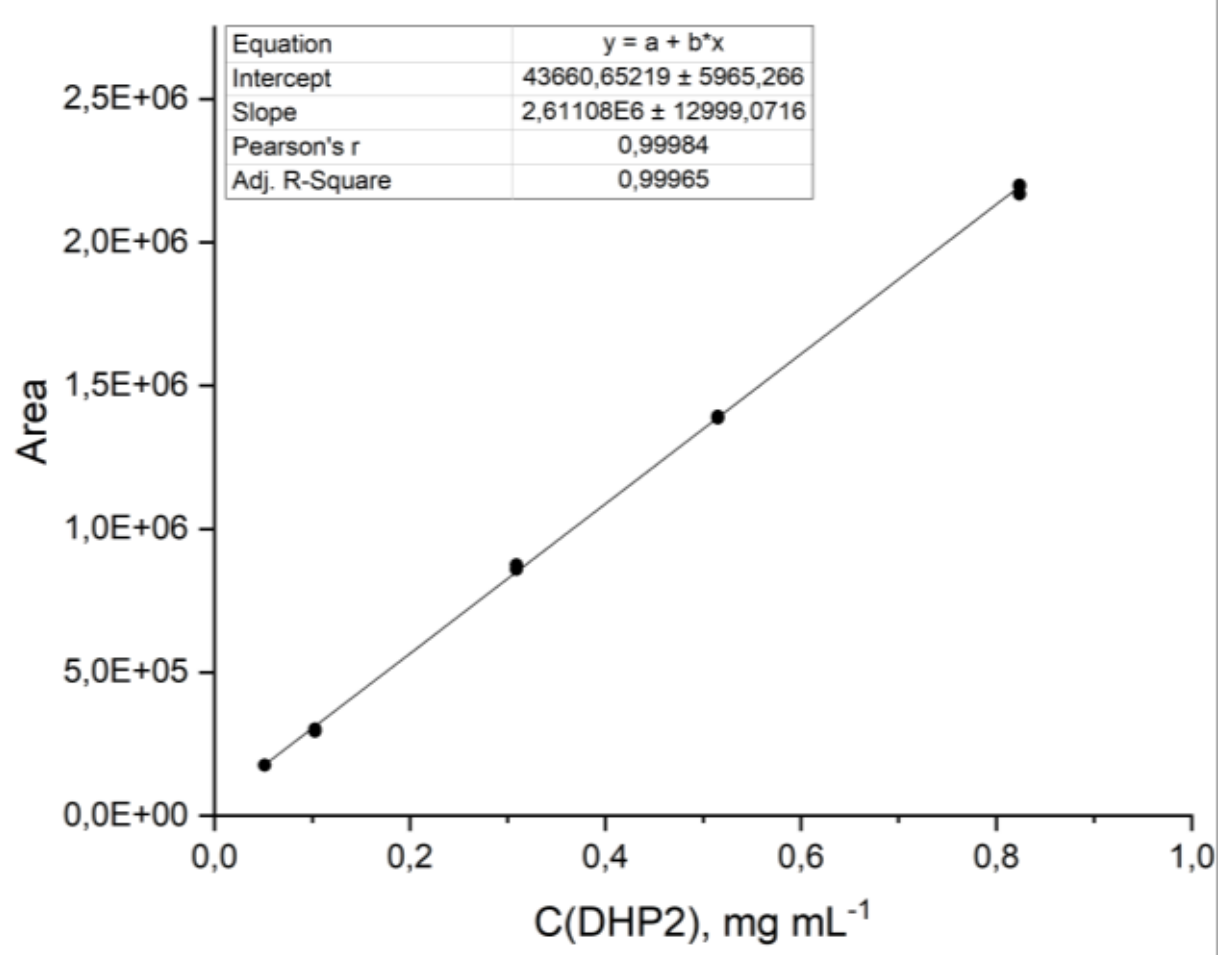

Figure S2: Calibration curve for DHP2.

Table S2: Time influence to CB7:DHP2 complexation.

Object	Time interval				
	30 min	90 min	3 h	24 h	72 h
	amount of DHP2 in water (by HPLC), %				
pure DHP2	21.6	16.3	20.1	13.1	13.4
1:1 molar ratio of CB7:DHP2	63.8	65.6	81.2	61.8	63.9
2:1 molar ratio of CB7:DHP2	83.2	90.7	90.6	87.1	88.6

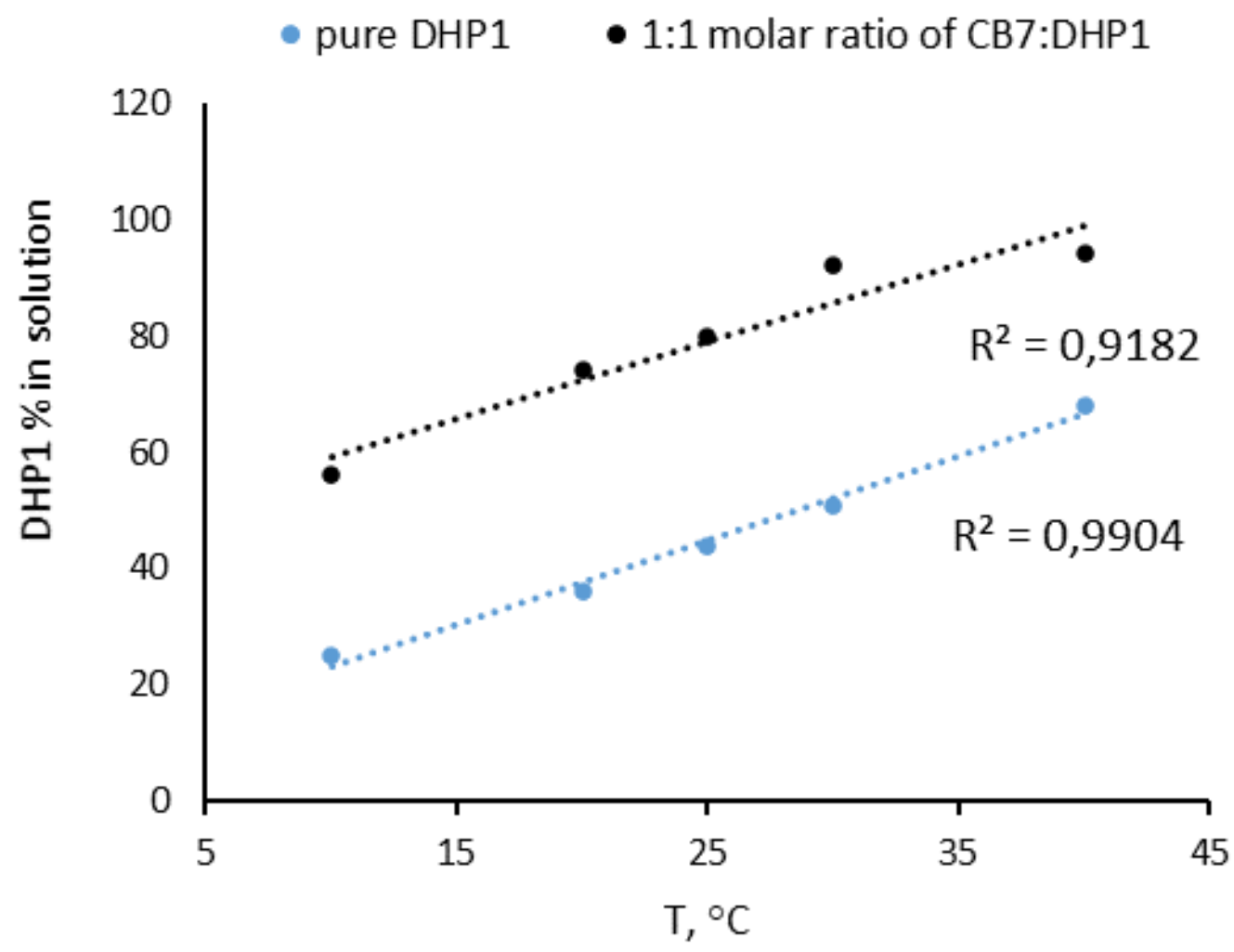

Figure S3: Solubility changes with temperature increasing for pure DHP1 and CB7:DHP1 (molar ratio of 1:1).

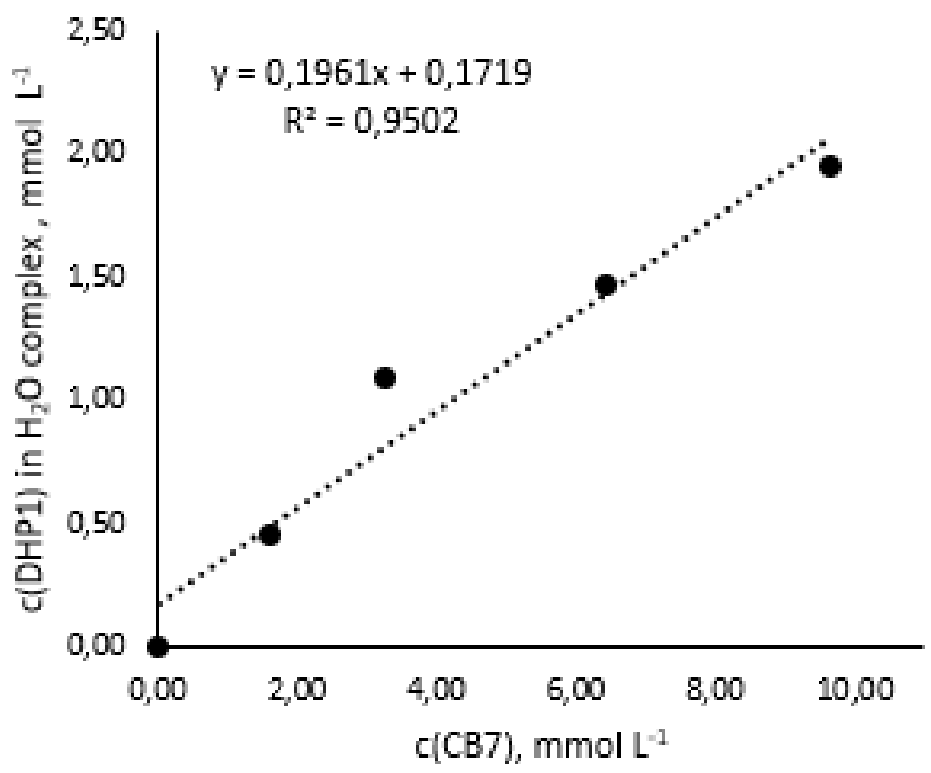

Figure S4: The illustration of the DHP1 concentration dependence from the CB7 amount in water.

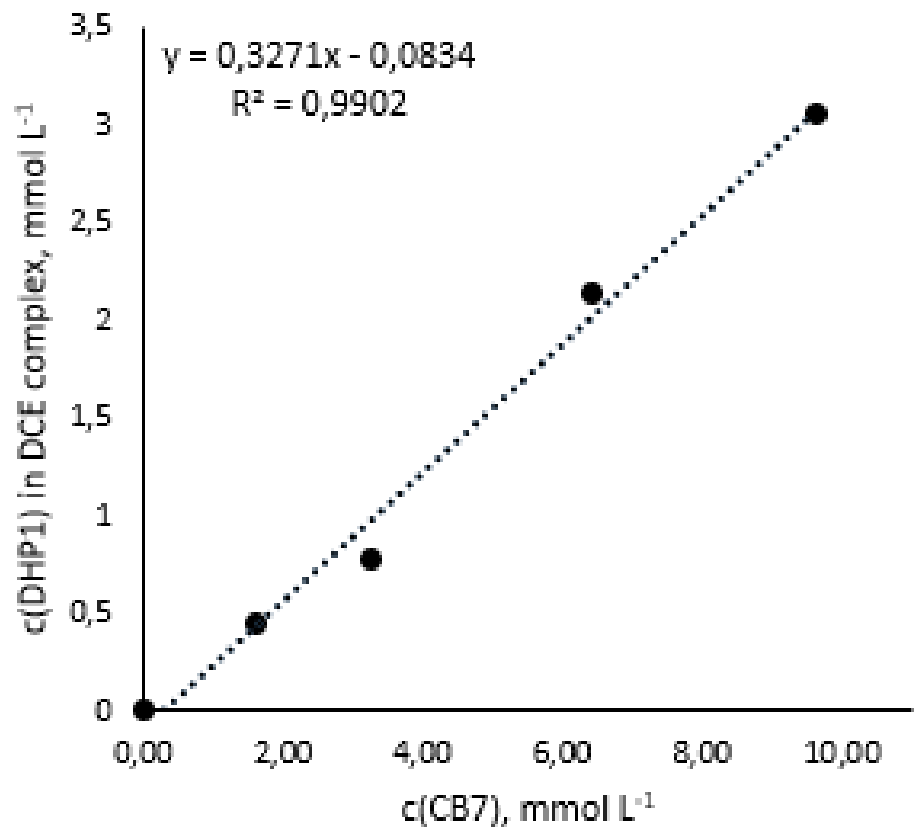

Figure S5: The illustration of the DHP1 concentration dependence from the CB7 amount in DCE.

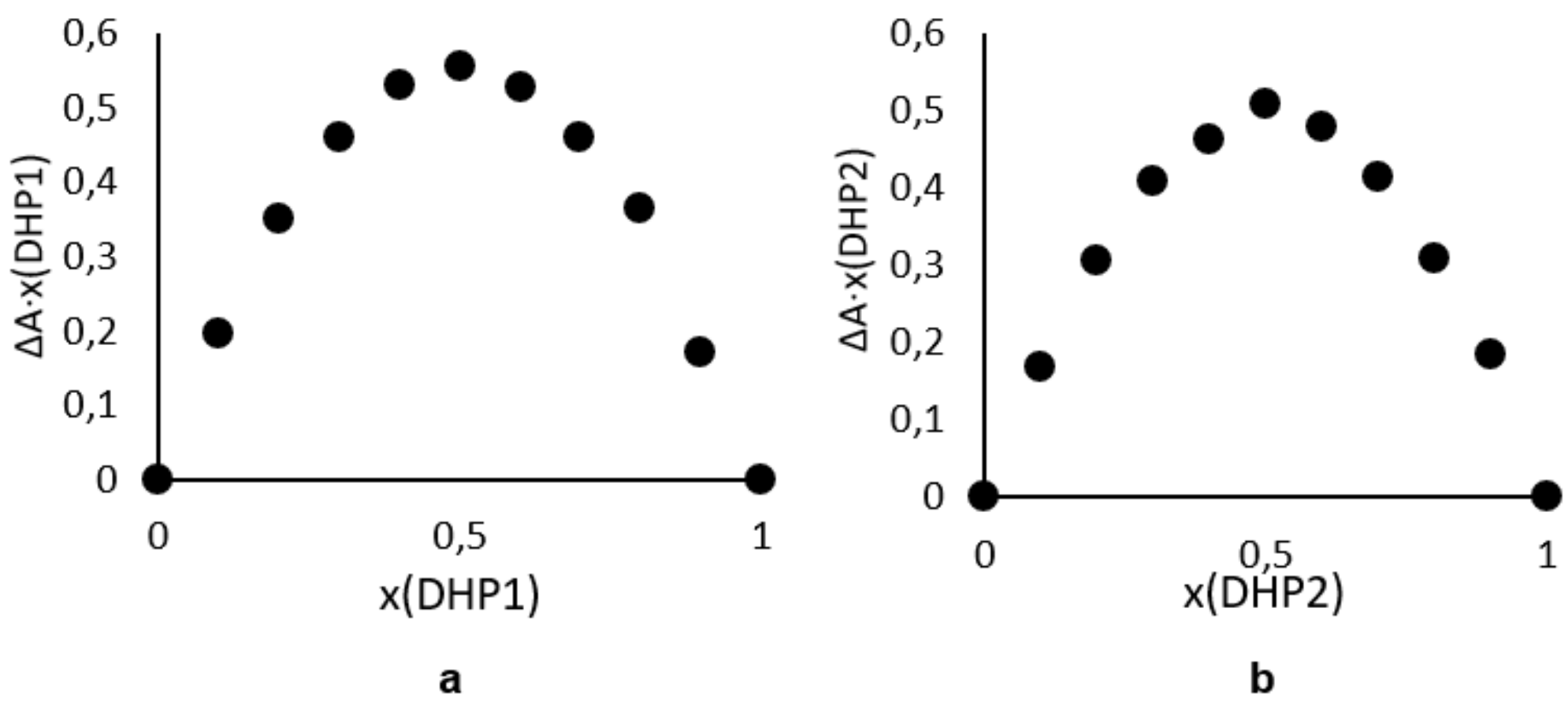

Figure S6: Job`s plots for CB7:DHP1 (a) and CB7:DHP2 (b).

NMR and MS spectra

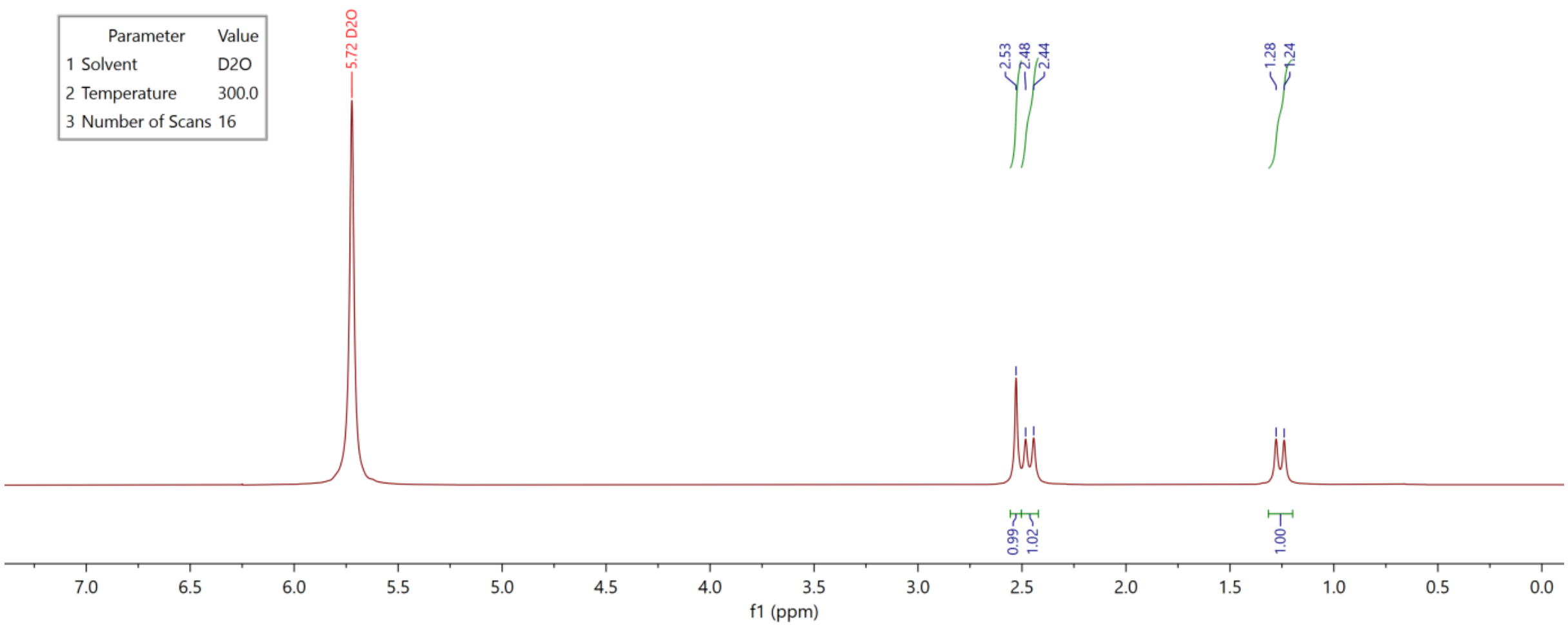

Figure S7: ^{1}H NMR spectrum of CB7 (35 wt % DCl in D_2O).

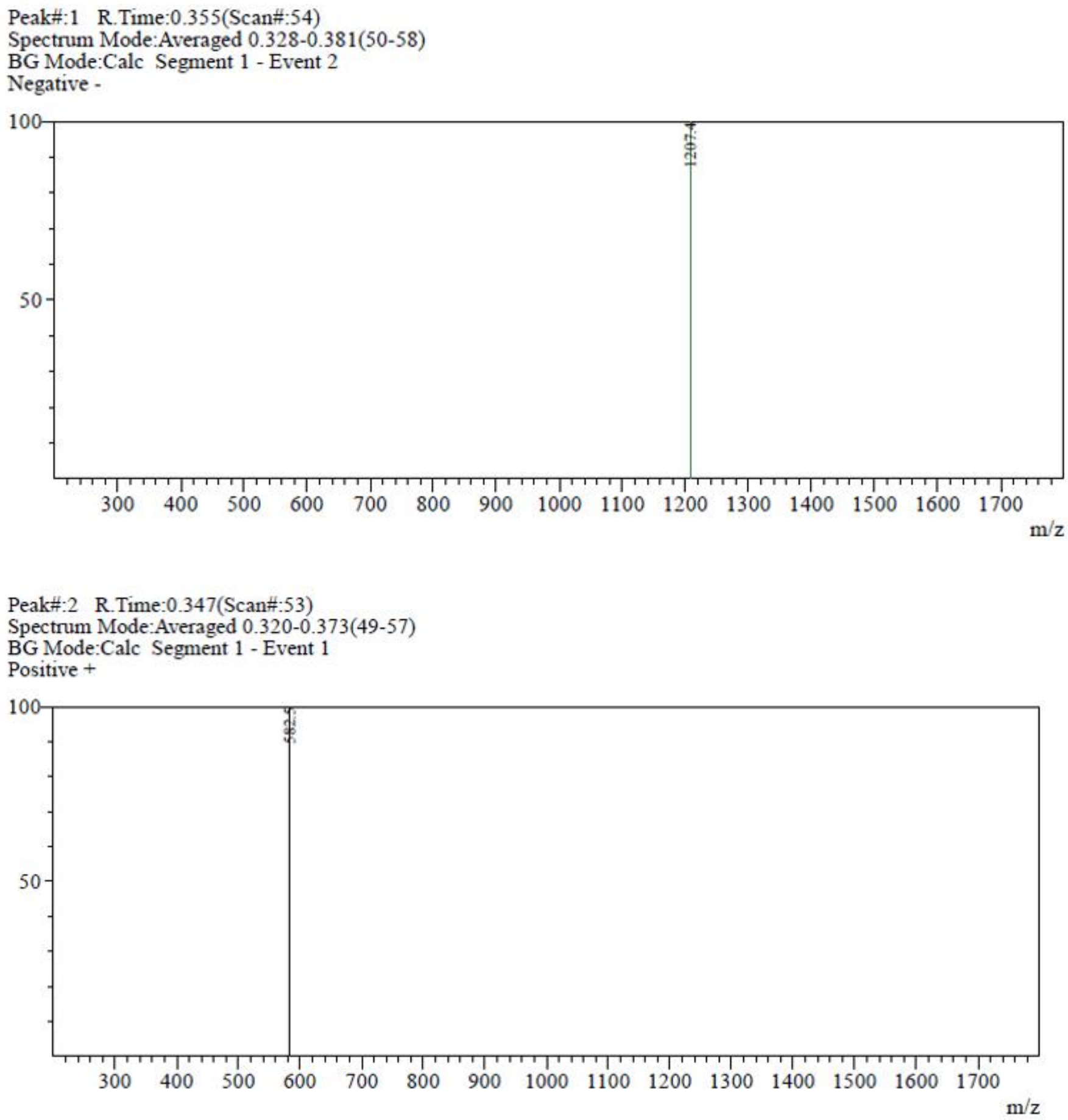

Figure S8: ESIMS spectrum of CB7.

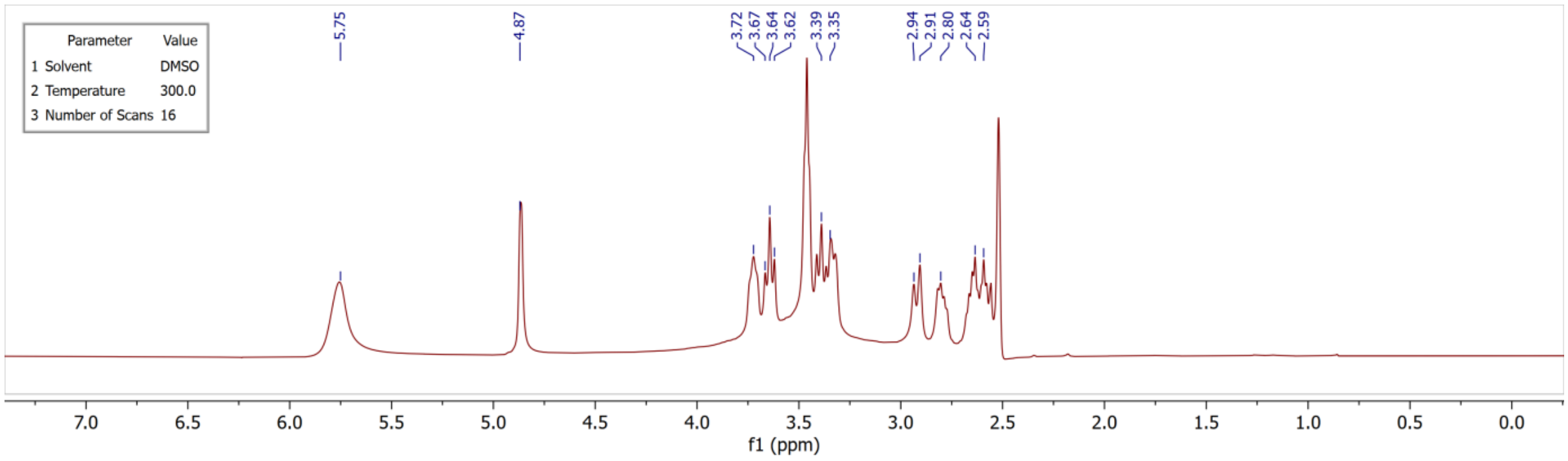

Figure S9: ^{1}H NMR spectrum of HEA-β-CD (DMSO-d_6).

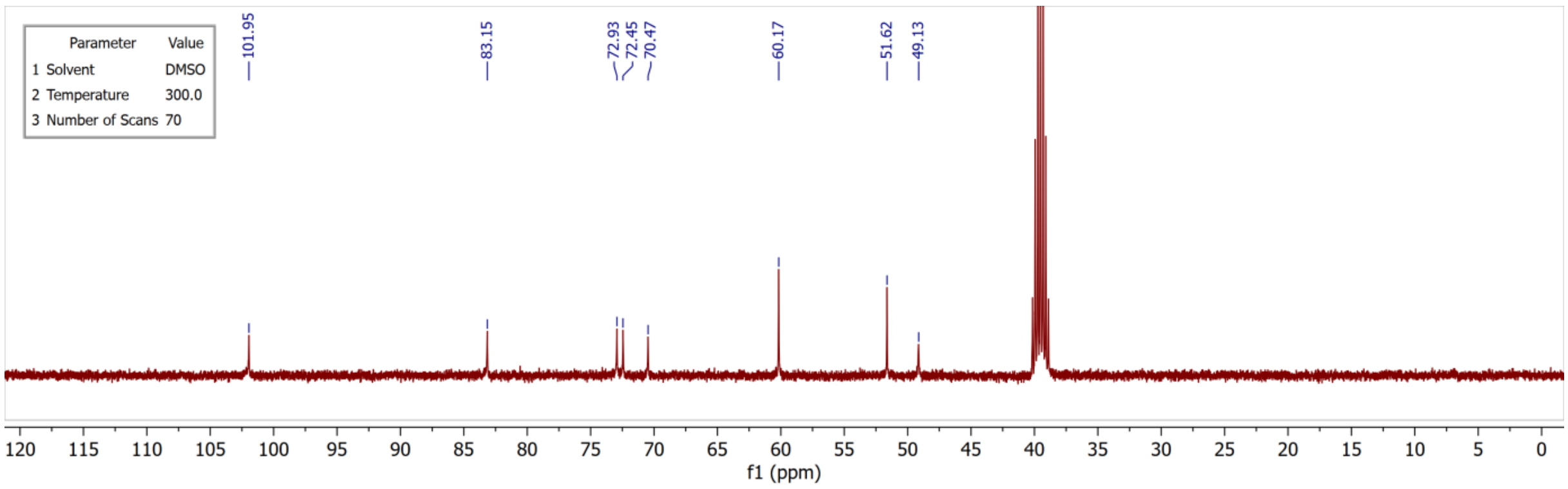

Figure S10: ^{13}C NMR spectrum of HEA-β-CD (DMSO-d_6).

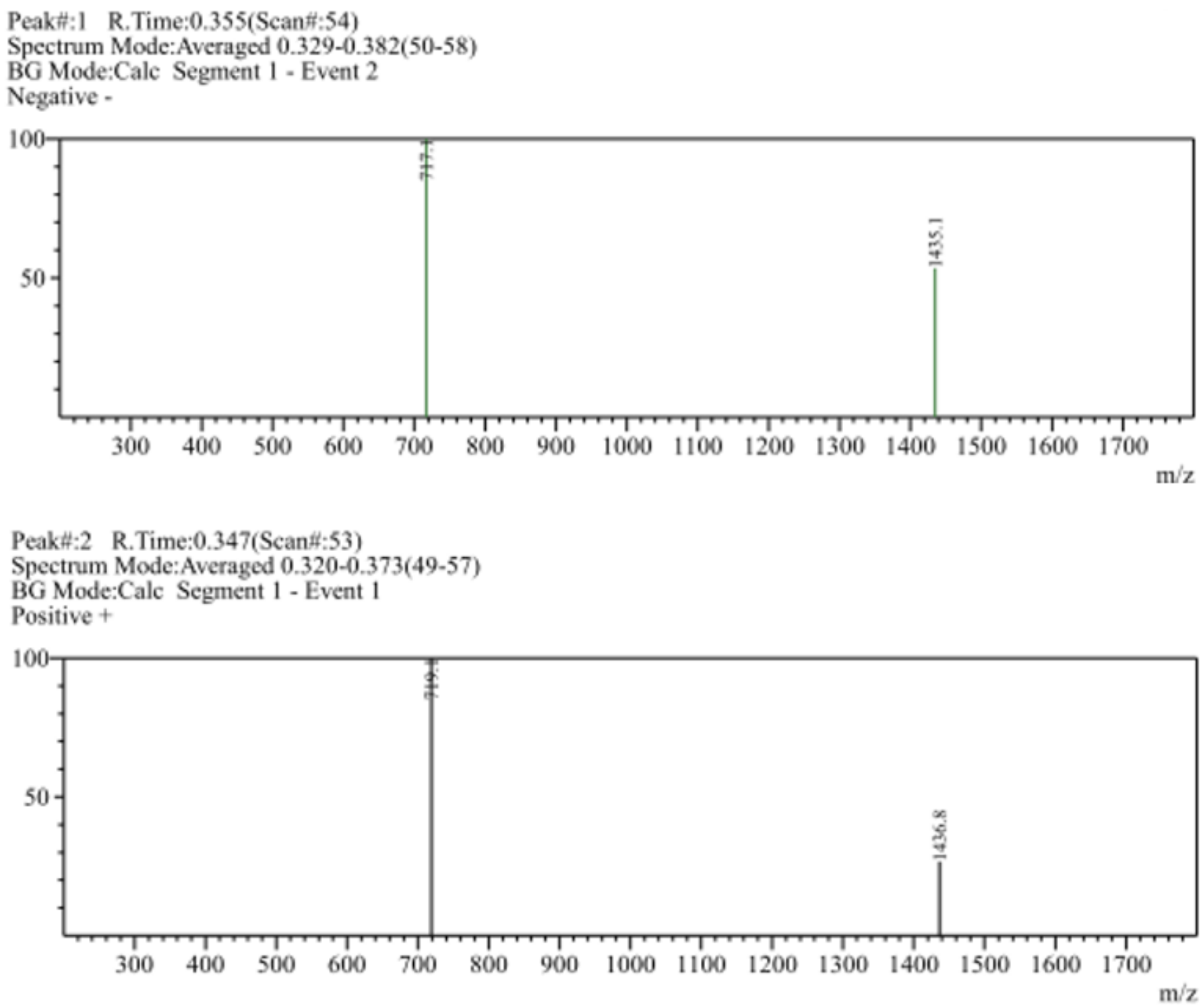

Figure S11: ESIMS spectrum of HEA-β-CD.

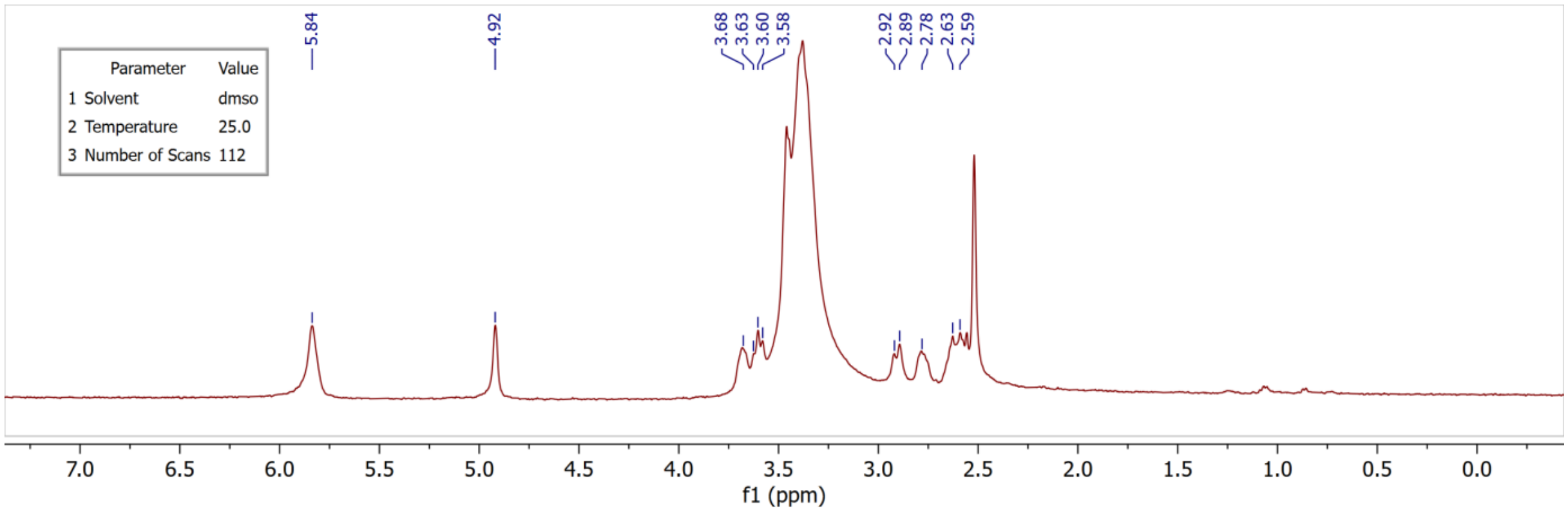

Figure S12: ^{1}H NMR spectrum of HEA-γ-CD (DMSO-d_6).

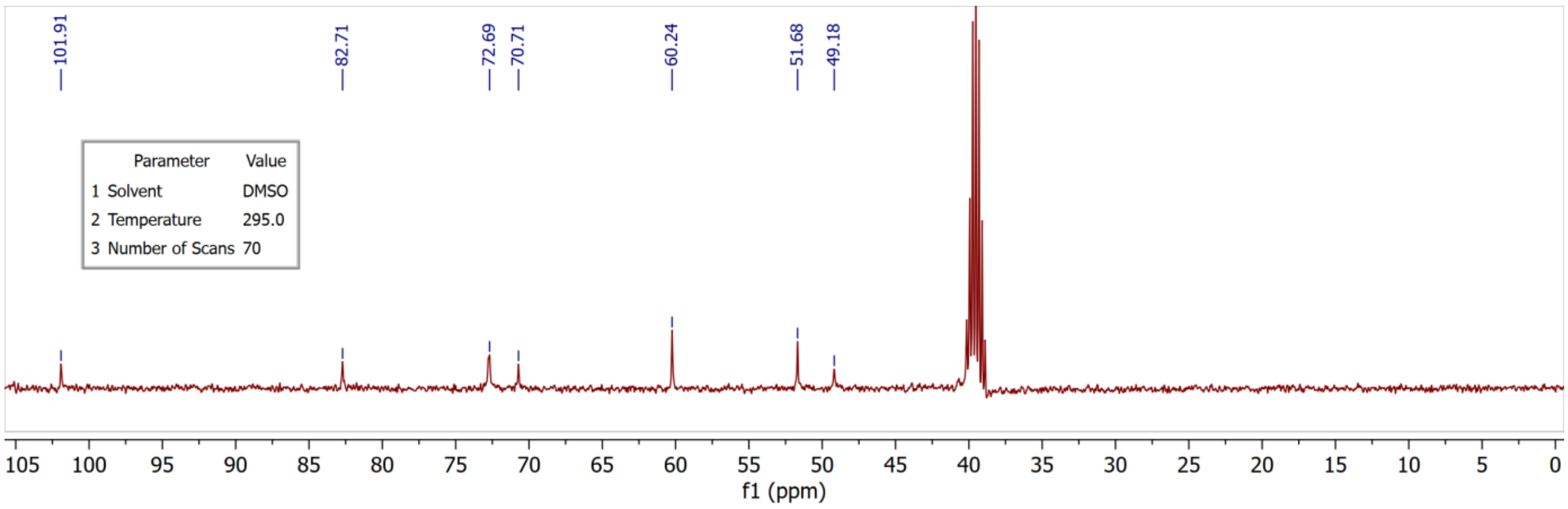

Figure S13: ^{13}C NMR spectrum of HEA-γ-CD (DMSO-d_6).

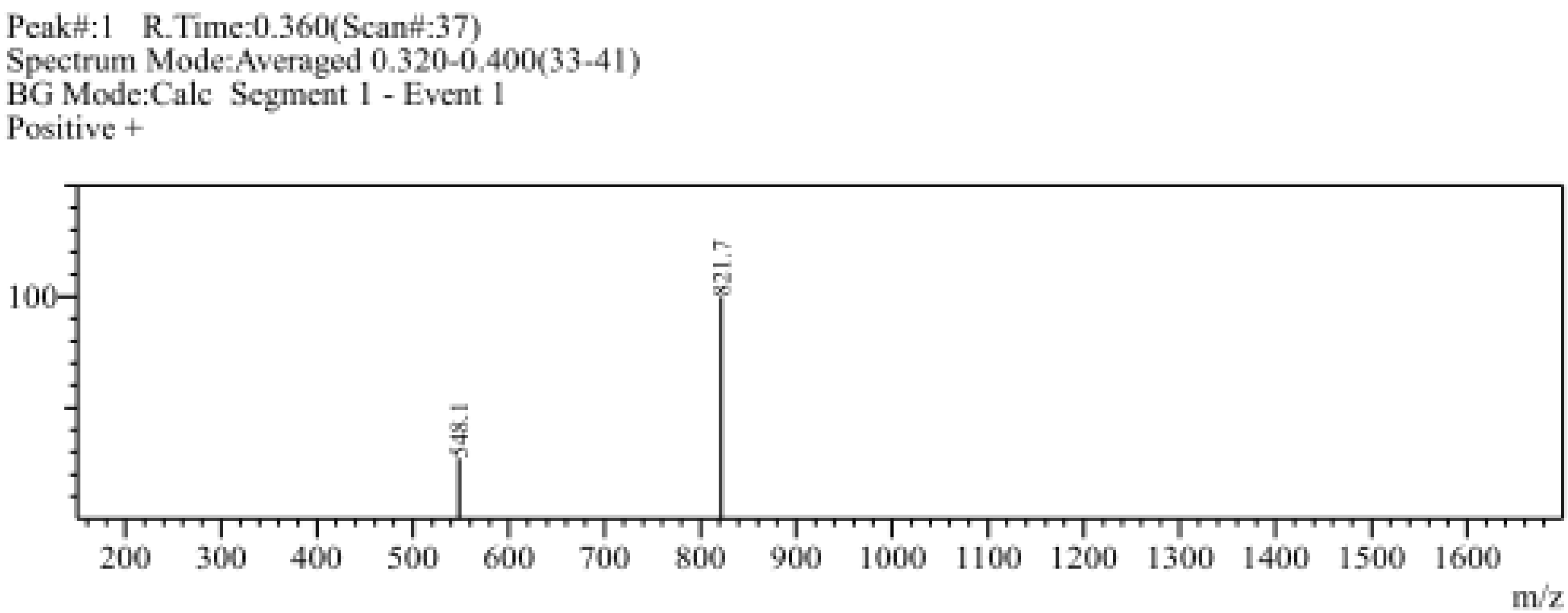

Figure S14: ESIMS spectrum of HEA-γ-CD.

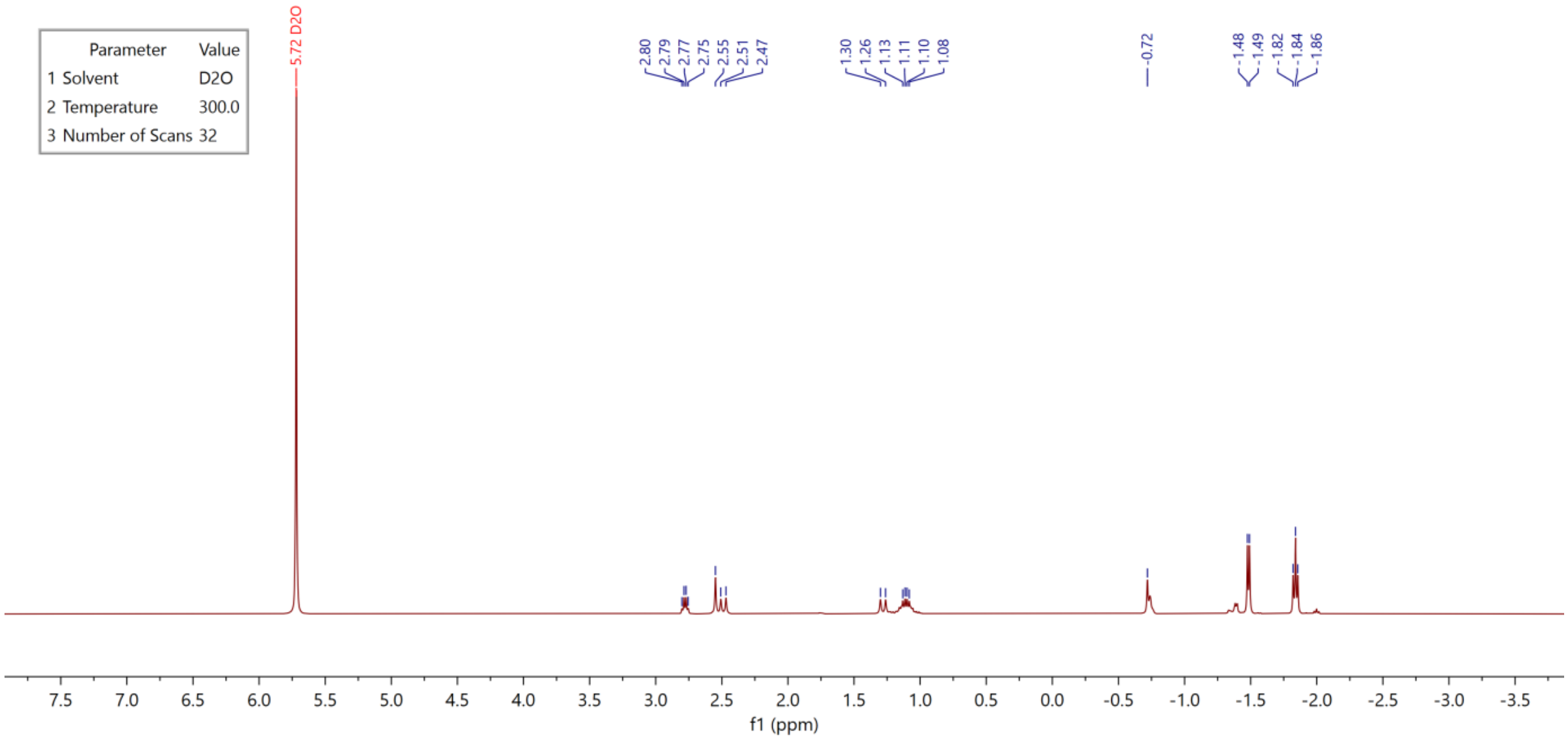

Figure S15: ^{1}H NMR spectrum of CB7:DHP1 (35 wt % DCl in D_2O).

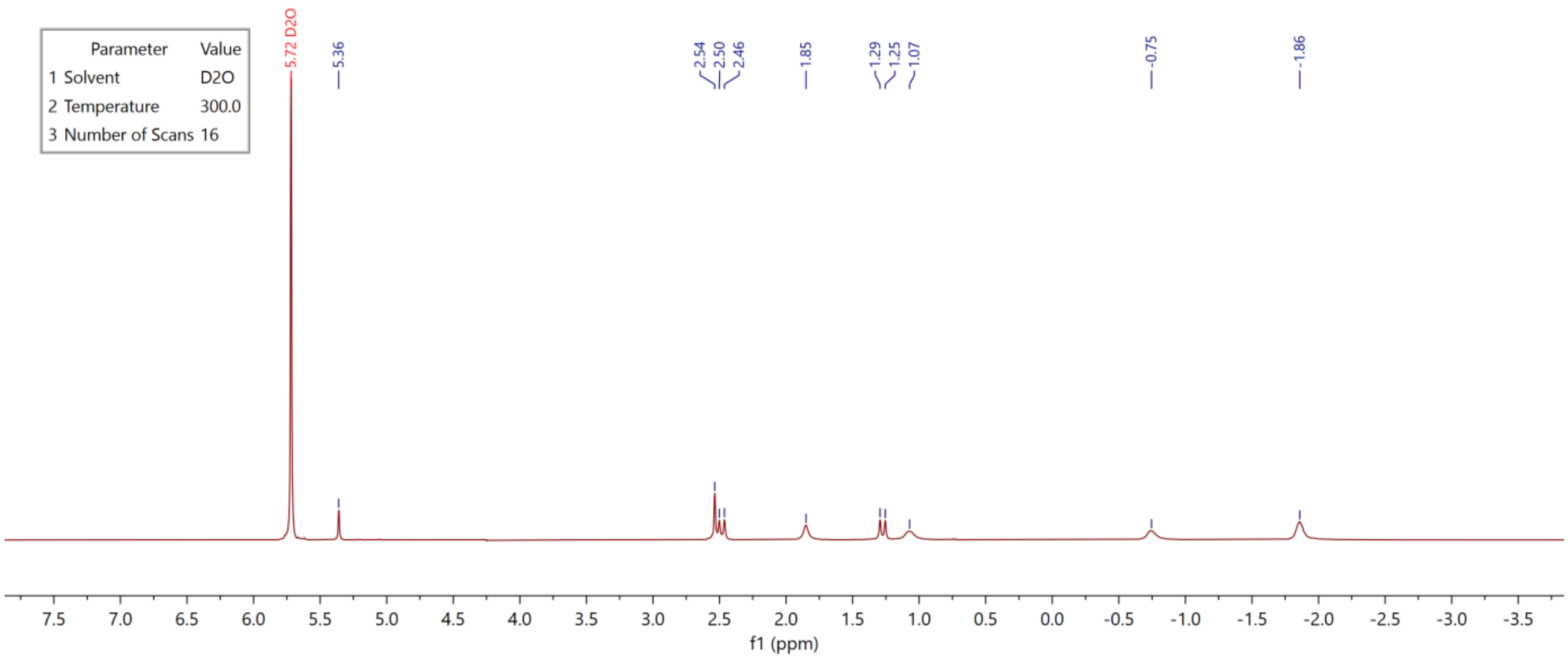

Figure S16: ^{1}H NMR spectrum of CB7:DHP2 (35 wt % DCl in D_2O).

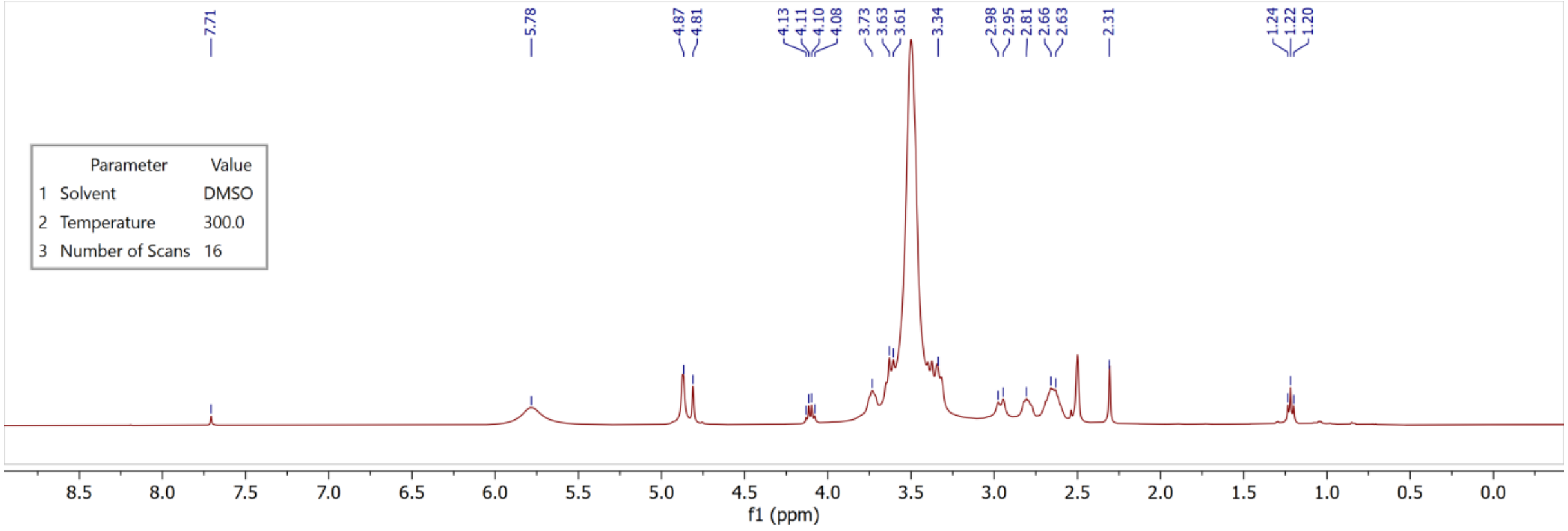

Figure S17: ^{1}H NMR spectrum of HEA-β-CD complex with DHP2 (DMSO-d_6).

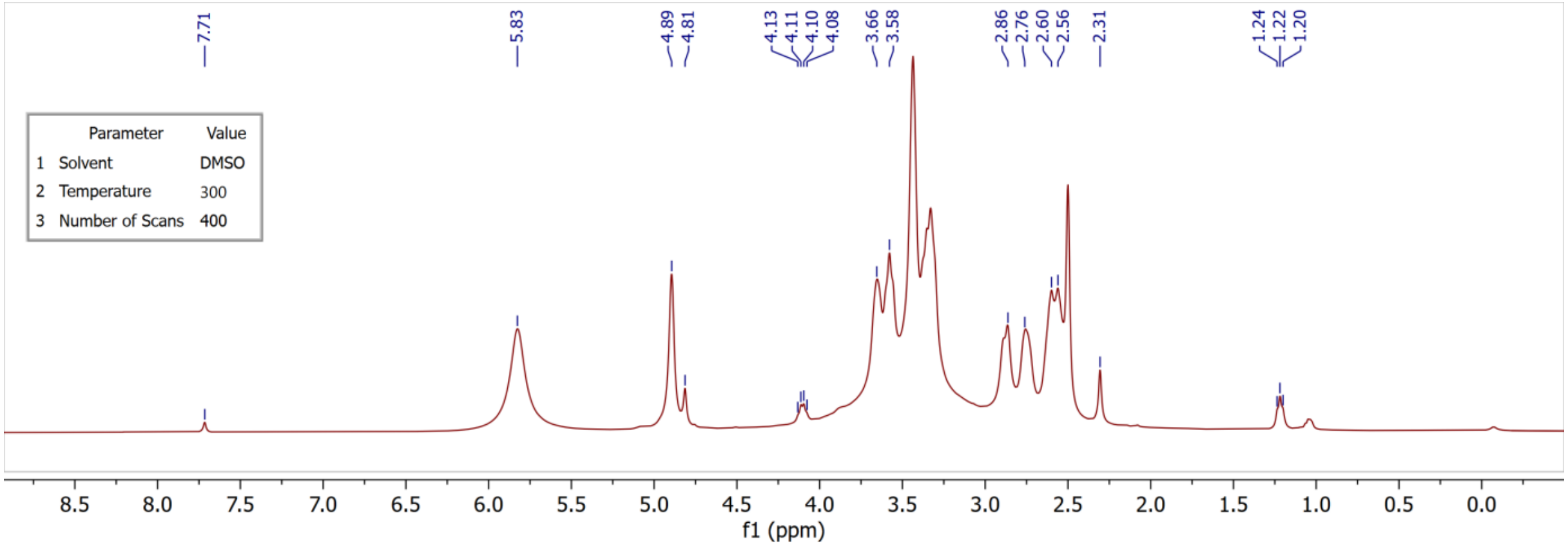

Figure S18: ^{1}H NMR spectrum of HEA-γ-CD complex with DHP2 (DMSO-d_6).

References

(1) Kolosov, M. A.; Shvets, E. H.; Manuenkov, D. A.; Kulyk, O. G.; Mazepa, A. V; Orlov, V. D. *Synth. Commun.* **2019**, *49*, 611–615. doi:https://doi.org/10.1080/00397911.2019.1566476

(2) Tkachenko, I. G.; Komykhov, S. A.; Musatov1, V. I.; Shishkina, S. V.; Dyakonenko, V. V.; Shvets, V. N.; Diachkov, M. V.; Chebanov, V. A.; Desenko, S. M. *Beilstein J. Org. Chem.* **2019**, *15*, 2390–2397. doi:10.3762/bjoc.15.231

(3) Komykhov, S. A.; Tkachenko, I. G.; Musatov, V. I. *Arkivoc* **2016**, *2016*, 277–287. doi:http://dx.doi.org/10.3998/ark.5550190.p009.610

(4) Miasnikova, D.; Kyrychenko, A. V; Vashchenko, O. V; Mateychenko, P. V; Pinchukova, N. A.; Musatov, V. I.; Chebanov, V. A. *J. Mol. Liq.* **2025**, *435*, 128143. doi:10.1016/j.molliq.2025.128143

(5) Day, A. I.; Arnold, A. P. Cucurbiturils and Method for Synthesis. WO 00/68232, 16 November 2000

(6) Lagona, J.; Mukhopadhyay, P.; Chakrabarti, S.; Isaacs, L. *Angew. Chemie - Int. Ed.* **2005**, *44*, 4844–4870. doi:10.1002/anie.200460675

(7) Davuluri, R. R.; Ponnaiah, R.; Sribhashyam, R. K.; Mecherili Valsan, N.; Dongari, N.; Chlnv, P. Improved Process for Preparation of Sugammadex. WO Patent 2012025937, 01 March 2012

(8) Chouker, M. A.; Abdallah, H.; Zeiz, A.; El-Dakdouki, M. H. *J. Mol. Struct.* **2021**, *1235*, 130273. doi:10.1016/j.molstruc.2021.130273

(9) Waleczek, K. J.; Cabral Marques, H. M.; Hempel, B.; Schmidt, P. C. *Eur. J. Pharm. Biopharm.* **2003**, *55*, 247–251. doi:10.1016/S0939-6411(02)00166-2

(10) Veiga, F.; Fernandes, C.; Maincent, P. *Drug Dev. Ind. Pharm.* **2001**, *27*, 523–532. doi:10.1081/DDC-100105177

(11) Mendes, C.; Buttchevitz, A.; Kruger, J.; Bernardi, L. S.; Oliveira, P. R.; Silva, M. *Anal. Sci.* **2015**, *31*, 1083. – 1089
